# Genome-wide methylation patterns in Marfan syndrome

**DOI:** 10.1186/s13148-021-01204-4

**Published:** 2021-12-11

**Authors:** Mitzi M. van Andel, Maarten Groenink, Maarten P. van den Berg, Janneke Timmermans, Arthur J. H. A. Scholte, Barbara J. M. Mulder, Aeilko H. Zwinderman, Vivian de Waard

**Affiliations:** 1 Department of Cardiology, Amsterdam UMC, University of Amsterdam, Meibergdreef 9, 1105 AZ Amsterdam, The Netherlands; 2 Department of Radiology, Amsterdam UMC, Amsterdam, The Netherlands; 3 Department of Cardiology, University Medical Center Groningen, University of Groningen, Groningen, The Netherlands; 4 Department of Cardiology, Radboud University Hospital, Nijmegen, The Netherlands; 5 Department of Cardiology, Leiden University Medical Center, Leiden, The Netherlands; 6 Department of Clinical Epidemiology, Biostatistics and Bioinformatics, Amsterdam UMC, Amsterdam, The Netherlands; 7 Department of Medical Biochemistry, Amsterdam UMC, Amsterdam Cardiovascular Sciences, Amsterdam, The Netherlands

**Keywords:** Marfan syndrome, EWAS, Methylation loci, Aortic diameters, Clinical events

## Abstract

**Background:**

Marfan syndrome (MFS) is a connective tissue disorder caused by mutations in the Fibrillin-1 gene (FBN1). Here, we undertook the first epigenome-wide association study (EWAS) in patients with MFS aiming at identifying DNA methylation loci associated with MFS phenotypes that may shed light on the disease process.

**Methods:**

The Illumina 450 k DNA-methylation array was used on stored peripheral whole-blood samples of 190 patients with MFS originally included in the COMPARE trial. An unbiased genome-wide approach was used, and methylation of CpG-sites across the entire genome was evaluated. Additionally, we investigated CpG-sites across the FBN1-locus (15q21.1) more closely, since this is the gene defective in MFS. Differentially Methylated Positions (DMPs) and Differentially Methylated Regions (DMRs) were identified through regression analysis. Associations between methylation levels and aortic diameters and presence or absence of 21 clinical features of MFS at baseline were analyzed. Moreover, associations between aortic diameter change, and the occurrence of clinical events (death any cause, type-A or -B dissection/rupture, or aortic surgery) and methylation levels were analyzed.

**Results:**

We identified 28 DMPs that are significantly associated with aortic diameters in patients with MFS. Seven of these DMPs (25%) could be allocated to a gene that was previously associated with cardiovascular diseases (HDAC4, IGF2BP3, CASZ1, SDK1, PCDHGA1, DIO3, PTPRN2). Moreover, we identified seven DMPs that were significantly associated with aortic diameter *change* and five DMP’s that associated with clinical events. No significant associations at *p* < 10^–8^ or *p* < 10^–6^ were found with any of the non-cardiovascular phenotypic MFS features. Investigating DMRs, clusters were seen mostly on X- and Y, and chromosome 18–22. The remaining DMRs indicated involvement of a large family of protocadherins on chromosome 5, which were not reported in MFS before.

**Conclusion:**

This EWAS in patients with MFS has identified a number of methylation loci significantly associated with aortic diameters, aortic dilatation rate and aortic events. Our findings add to the slowly growing literature on the regulation of gene expression in MFS patients.

**Supplementary Information:**

The online version contains supplementary material available at 10.1186/s13148-021-01204-4.

## Introduction

Marfan syndrome (MFS) is a dominant autosomal disorder caused by mutations in the Fibrillin-1 (FBN1) gene, which results in a connective tissue defect. FBN1 is a relatively large gene consisting of 65 exons that encodes the proprotein profibrillin-1, consisting of 2871 amino-acids, which is proteolytically cleaved into fibrillin-1 and the hormone asprosin. Over 3000 pathogenic variations have been reported [1]. MFS has diverse phenotypic expression covering skin, lungs, eyes, joints, bones, the spinal cord and in the cardiovascular system. Mitral valve prolapse and aortic aneurysms in the heart and the aorta, respectively, determine the increased risk for cardiovascular morbidity and mortality.

There is large phenotypic variation, also within families with members carrying the same genetic mutation. Different patient-characteristics have been evaluated as explanatory factors for the phenotypic diversity. The multitude in FBN1-mutations, leading to for example defining dominant negative or haploinsufficient FBN1-expression phenotypes, have been implicated in disease severity and response to pharmaceutical treatments [2–6]. An altered distribution of FBN1 transcript isoforms has been identified between MFS patients and unaffected individuals [7]. Moreover, the abundance of the wild-type allele expression of the FBN1 gene may contribute to disease severity [8]. Also, genetic variations in other genes at other chromosomal loci have been reported to be associate with enhanced aorta pathology MFS, such as COL4A1 and PRKG1, which are considered genetic modifiers [9]. It is anticipated that variants in more aneurysm-related genes will be discovered to affect MFS phenotype.

Association of increased blood levels of elastin fragments (desmosine), transforming growth factor beta (TGF-β), microfibrillar associated protein 4 (MFAP4), or homocysteine with enhanced aorta pathology is described in MFS [10–13].

Recently, DNA-hypomethylation patterns of CpG-island shores of the FBN1-gene were associated with the level of FBN1 gene expression in porcine liver and fetal fibroblasts, showing its involvement in tissue/cell-type-specific gene expression [14]. To our knowledge, no other studies concerning the role of methylation patterns in MFS patients have been performed yet. Differential methylation patterns of the FBN1 locus have been observed; however in other patient populations, hypermethylation of the FBN1 gene is identified as biomarker for multiple cancers [15–17]. Although it is unclear how these results generalize to MFS-patients, it illustrates that differential methylation patterns impact FBN1-expression and may thus be associated with MFS phenotypic diversity.

With the current study we described the association between a snapshot of the genome-wide DNA-methylation levels and different MFS phenotypes in a subgroup of the patients who were included in the COMPARE trial on the effect of losartan [18]. DNA-methylation was measured using the Illumina 450 K chip with DNA derived from stored peripheral whole-blood samples (taken at baseline) of 190 participants. We used an unbiased genome-wide approach and thus evaluated methylation of CpG-sites across the entire genome. Because MFS is caused by mutations in the FBN1 gene, we also looked in particular at CpG-sites up- and downstream from the FBN1-locus (15q21.1).

## Methods

### Study population

This study is part of the COMPARE study on efficacy of losartan treatment to reduce growth of the aortic root and thoracic aorta diameter in patients with MFS. Details of the design and data collection [19] are described elsewhere as well as of the primary results [18]. In brief, the COMPARE study was a multicenter randomized clinical trial randomizing 233 patients with MFS between losartan 50 and 100 mg daily on top of usual treatment versus usual treatment for 3 years. MFS was established according to the revised Ghent criteria, all patients were > 18 years of age, had diameters < 50 mm at baseline, did not have > 1 surgical repair of aortic aneurysms and were not scheduled for surgery. Diameters of the aortic root and thoracic aorta were measured at seven anatomical locations at baseline and after 3 years, by Magnetic Resonance Imaging (MRI) or by Computed Tomography (CT) imaging. Losartan was found to significantly reduce growth of the diameter of the aortic root. Ethical approval was obtained from the ethics committees of all hospitals from which patients were included. Written informed consent was obtained from all study patients.

The methylation-wide association study was performed using a subset of all 194 patients of whom stored whole-blood was available from baseline.

### Phenotypic measurements

*Primary outcomes* for the present study were the baseline diameters of the aorta, measured at seven anatomical aortic landmarks. Aortic diameters were measured on the MRI and CT scans; the aortic root, the ascending and descending thoracic aorta at the level of the pulmonary bifurcation, the aortic arch, the descending thoracic aorta at the level of the diaphragm and the abdominal aorta just proximal to the aortic bifurcation. See Groenink et al. [18] for a detailed description of MRI, and CT acquisitions. Aortic root diameter was assessed by greatest end-diastolic diameter of three cusp-cusp dimensions from the outer to inner wall on the steady-state free-precession images. All measurements beyond the aortic root were performed on multiplanar magnetic resonance angiography reconstructions from inner to inner edge.

*Secondary outcomes* were presence or absence at baseline of 21 clinical features of MFS: ectopia lentis, myopia, flat cornea, increased axial eye-length, hypoplastic iris, thumb/wrist sign, pectus carinatum, pectus excavatum, hindfoot deformity, pes planus, pneumothorax, dural ectasia, protrusio, increased height-span ratio, scoliosis, reduced elbow extension, facial features, striae, joint problems, arched palate, and mitral valve prolapse. We also evaluated the Marfan disease systemic score [20], which is a weighted sum of the mentioned MFS clinical features. Presence or absence of the MFS features was taken from electronic patient files.

Moreover, we evaluated the association of the changes in aortic diameter with methylation levels during the 3-year trial. Change was calculated as the absolute difference between diameters measured after the 3 years follow-up of the trial minus the diameters measured at baseline.

Lastly, we analyzed the association between methylation levels and the occurrence of clinical events (death any cause, type-A or -B dissection/rupture, or aortic surgery) over a median follow-up of 8 years [21].

Missing values were multiply imputed and all results that we report below were based on observed and imputed values. We performed a sensitivity analysis based on observed values only, and these results were similar to what was found with observed and imputed data (Additional file 1: Figure SA1). Distributions of the baseline diameters and their changes were normally distributed within a reasonable range in our patient samples, but a few large diameters were observed at the diaphragm and bifurcation, and were characterized as outliers (Additional file 1: Figures SB1 and SB2). Baseline diameters were substantially correlated with each other (varying from 0.21 to 0.79), and their first principal component explained about 57% the total variance in the diameters at the seven landmarks. We also evaluated therefore the association between methylation levels and the mean diameter calculated over the seven landmarks, but this did not add any new insights.

### DNA methylation profiling and processing

DNA extraction and methylation profiling were performed on whole-blood, available from baseline (2008–2009). Bisulfite DNA treatment was achieved using the Zymo EZ DNA MethylationTM kit, and the quality of the conversion was determined by high-resolution melting analyses. The converted DNA was amplified and hybridized on the Illumina Human Methylation 450 K array, which measured DNA methylation levels of approximately 485,000 CpG sites. The samples were randomly divided over three bisulfite conversion and hybridization batches.

Raw 450 K data were processed for primary quality control using the statistical language R (version 4.0.3) and various packages (methylAid, minfi). Bad quality samples were detected using sample dependent and the sample-independent control CpG sites present on the 450 K array itself. Threshold values for defining bad quality samples were: methylated and unmethylated intensities of 10.5, overall quality control = 11.75, bisulfite control = 12.75, hybridization control of 12.50, and detection *p* value of 0.95. Based on these thresholds, four samples were considered outliers (Additional file 1: Figure SC1). This resulted in a sample size of 190 for the current analyses.

Functional normalization was applied using the preprocess Funnorm function of the R-minfi package to normalize raw 450 K data. Principal component analysis (PCA) on the normalized dataset annotated for sex, age, body surface area, recruitment site, bisulfite batch, hybridization batch and plate position revealed no other quality concerns (Additional file 1: Figure SC2). Principal components 1 and 2 explained 30% of the variance.

We discarded CpG sites referring to single nucleotide polymorphisms and single base extensions, and this resulted in a set of 467,971 CpG sites which was used to identify differentially methylated positions (DMP) and differentially methylated regions (DMRs) in the regression analyses. There were 4414 methylation measurements with detection *p* values larger than 0.05 (0.0048%), and for 463,557 CpG sites all detection *p* values were smaller than 0.05. Per patient, the number of CpG sites with methylation detection *p* values > 0.05 varied between 9 and 1117 with median 271 (Q1–Q3: 61–259).

Cell composition of the whole-blood samples was estimated using the method proposed by Houseman et al. [22]. Cell-type distribution variables were used as covariates in the regression analyses: percentage of CD8^+^ T-cells, CD4^+^ T-cells, B-cells, NK-cells, Monocytes, Neutrophils and Eosinophils.

### Statistical analysis

#### Differentially methylated positions (DMPs)

Linear, logistic and Cox regression analyses were performed in R. Aorta diameters, their change, MFS features and the occurrence of events were the dependent variables. DNA methylation M-levels were the independent variables of main interest. Age, sex, body surface area (BSA), estimated cell counts were included as covariates. The methylation principal components had high multivariate correlation with the cell-type distribution variables (first canonical correlation 0.9927) and were therefore not included as covariates. Because of the low incidence, we only performed univariate Cox regression analysis on the combined clinical events.

For all DMP analyses, M-values were calculated as the log2 ratio of the intensities of methylated probes versus unmethylated probes. The results of the regression analyses were presented as regression weights with corresponding standard errors and *p* values. We also analyzed the methylation Beta-values. Because Beta-values are logit-transformations of the M-values (times log(2)), results of the analyses with Beta-values were highly similar to those using M-values, and we focus therefore only on M-values.

#### Differentially methylated regions (DMRs)

To identify DMRs we smoothed per chromosome the estimated regression coefficients of the regression models of phenotypes on the 467,971 M-values inversely weighted by the associated squared standard errors. We used a loess-smoother with span-width 0.01 for M-values on all autosomes and 0.1 for M-values on the Y-chromosome.

We defined a DMR as three or more CpG sites in a cluster. Statistical significance of DMRs was obtained from bootstrapping, but because the choice of the span-width of the loess-smoother was based on subjective visual inspection only, significance levels should be interpreted with caution.

Gene-enrichment analysis of genes associated with identified DMPs or DMRs was performed with Panther (version 16.0) through geneontology.org and genemania.org.

## Results

### Description of the MFS patient population

Patient characteristics and disease phenotypes are summarized in Tables 1 and 2. Average age was 38 years (SD 13) and there were 103 male and 87 female patients.

**Table 1:** Baseline characteristics

	Total *n* = 190
*Patient characteristics*	
Sex, male	103 (54%)
Age, years	38 ± 13
Body surface area (m^2^)	2.02 ± 0.24
*Estimated cell fractions (%)*	
B cells	4 ± 3
NK cells	5 ± 4
CD4^+^ T cells	17 ± 6
CD8^+^ T cells	4 ± 3
Neutrophils	59 ± 10
Monocytes	10 ± 3
Eosinophils	0.1 ± 0.8

Plus–minus values are means ± SD

**Table 2:** (A) Aortic diameter and aortic dilatation rate by MRI. (B) Non-cardiovascular Marfan features

	Aortic diameters^a^	Aortic diameter change^b^
A		
*Aortic dimension by MRI*		
Aortic root (mm)	45 ± 5.6	1.23 ± 2.10
Ascending aorta (mm)	29 ± 3.9	0.84 ± 1.39
Aortic arch (mm)	24 ± 3.1	0.57 ± 1.47
Proximal descending aorta (mm)	24 ± 3.5	0.53 ± 1.54
Distal descending aorta (mm)	21 ± 3.1	0.81 ± 1.57
Diaphragm (mm)	21 ± 3.2	0.33 ± 1.21
Bifurcation	16 ± 3.6	0.46 ± 1.83

Marfan features		Marfan features	
B			
Ectopia Lentis	88 (46%)	Dural ectasia	98 (52%)
Myopia	37 (19%)	Protrusio	5 (3%)
Flat cornea	15 (8%)	Increased height-span ratio	37 (19%)
Increased axial eye-length	25 (13%)	Scoliosis	50 (26%)
Hypoplastic iris	17 (9%)	Reduced elbow extension	25 (13%)
Thumb/wrist signs	83 (44%)	Facial features	56 (29%)
Pectus carinatum	66 (35%)	Striae	128 (67%)
Pectus excavatum	29 (15%)	Joint problems	48 (25%)
Hindfoot deformity	56 (29%)	Arched palate	119 (63%)
Pes planus	83 (44%)	Mitral valve prolapse	104 (55%)
Pneumothorax	28 (15%)		

Summary statistics are based on observed values and imputations of missing values

^a^Data are diameters at baseline

^b^Data are change in millimeter per 3 years

Average baseline aortic diameters at the seven anatomical landmarks were 45, 29, 24, 24, 21, 21, and 16 mm, from aortic root to aortic bifurcation, respectively. Average increases of the diameters during the 3-year trial were 1.23, 0.84, 0.57, 0.53, 0.81, 0.33, and 0.46 mm, respectively. There were few missing baseline diameters at the bifurcation, because the MRI/CT images did not extend to that landmark. Baseline diameters of the root and ascending aorta were missing in 52 patients because of previous aortic surgery. A number of values for the change in diameter were missing because of aortic surgery during the 3-year follow-up of the trial. Prevalence of non-cardiovascular Marfan features varied between 3% (Protrusio) and 67% (Striae).

As expected, diameters showed significant positive correlation with age and BSA, and male patients had significantly larger diameters than female patients. Diameters also showed significant correlations with cell-type fractions, especially negatively with percentage of CD8^+^ T-cells (corr > − 0.22, *p* < 0.0022). There were 51 patients with clinical events during the 8 years follow-up. There were two aortic ruptures, 13 aortic dissections and 38 patients underwent aortic surgery. Five patients died (three after dissection/rupture, one had aortic surgery first). The Kaplan–Meier curve is given in Additional file 1: Figure SD1.

### Differentially methylated positions associated with aortic diameters

**Fig. 1:**
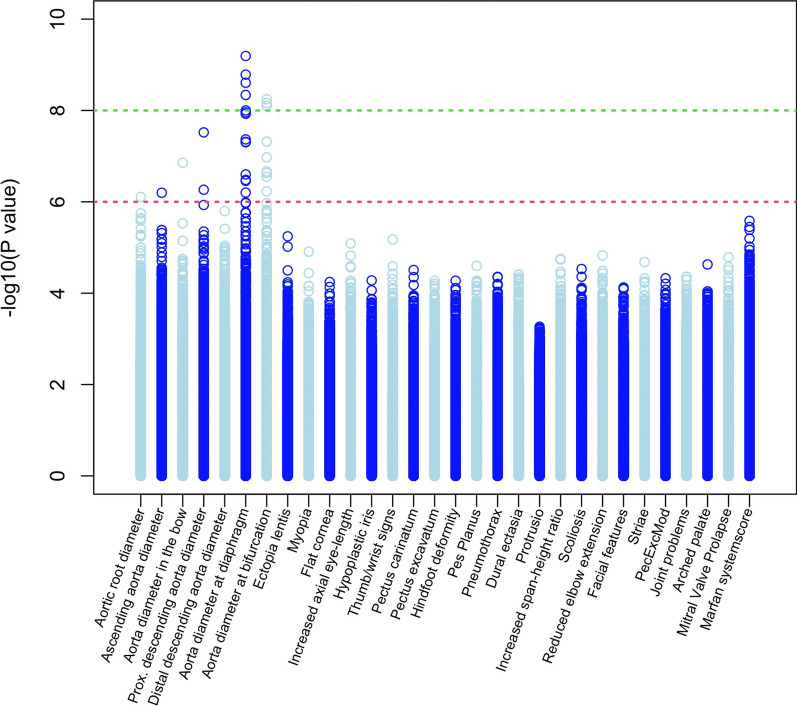
Minus ^10^log (*p* values) of the associations of methylation M-values and Beta-values at the 467.971 CpG sites with each of the baseline aortic diameters and Marfan features (so, every column consists of 2 * 467.971 *p* values). The dotted green and red lines correspond to 8.5 and 6 indicating *p* values of 8.5 × 10^–8^ and 1 × 10^–6^, respectively

We identified differentially methylated positions that are associated with aortic diameters. The *p* values of the regression analyses of all baseline aorta diameters and MFS features on all M-values corrected for sex, age, BSA, and cell-types are summarized in Fig. 1. In total there were eight CpG-sites with genome-wide significant association (*p* < 10^–8^) with baseline aortic diameters, at the diaphragm and bifurcation level (Table 3, bold). Of these eight CpG sites, six are close to, or in known genes.

**Table 3:** CpG-sites with genome-wide significant association with baseline aortic diameters (*p* < 10^–6^)

Aorta diameter	CpG	CHR	Position	Strand	Gene	UCSC_CpG_Islands_Name	*β* value	SE	*p* value	Cardiovascular function
Aortic root	cg20074307	14	55,092,491	F	SAMD4A		− 7.328698	1.42835322	7.81E−07	
Ascending aorta	cg25190999	2	8,826,175	F		chr2: 8825106–8826188	− 7.3255838	1.41517063	6.33E−07	
Aortic arch	cg13399952	9	132,652,889	F	FNBP1	chr9: 132652350–132652715	3.259245	0.59292635	1.39E−07	
Proximal desc aorta	cg22162225	2	62,932,835	R	EHBP1	chr2: 62932595–62933353	− 3.9168576	0.75191428	5.44E−07	
Proximal desc aorta	cg04258811	15	84,976,641	F		chr15: 84976070–84977044	2.73851672	0.47139499	3.02E−08	
Aorta at diaphragm	cg11825706	1	201,552,873	R			− 2.9891322	0.55625272	2.51E−07	
Aorta at diaphragm	cg10045864	2	240,036,897	F	HDAC4	chr2: 240033147–240033453	− 5.1346472	0.89881755	4.87E−08	Cardiac hypertrophy and remodeling
										Aortic aneurysm formation
										Vascular dysfunction
Aorta at diaphragm	cg18487516	6	3,849,542	F	FAM50B	chr6: 3849271–3851048	− 6.2306009	1.03917257	1.18E−08	
**Aorta at diaphragm**	**cg07829265**	**7**	**4,308,166**	**F**	**SDK1**	**chr7: 4303079–4305062**	− **3.9305036**	**0.62392327**	**2.47E−09**	**Hypertension**
Aorta at diaphragm	cg05852760	7	23,508,224	F	IGF2BP3	chr7: 23508184–23509712	− 3.0209111	0.52917492	4.97E−08	Congenital heart disease
Aorta at diaphragm	cg21375490	7	157,411,039	F	PTPRN2	chr7: 157409846–157410241	− 4.9468079	0.95539399	6.29E−07	Future myocardial infarction
**Aorta at diaphragm**	**cg07701530**	**10**	**22,911,629**	**F**	**PIP4K2A**		− **1.862081**	**0.29196299**	**1.64E−09**	
**Aorta at diaphragm**	**cg26558664**	**11**	**18,230,491**	**F**	**LOC494141**	**chr11: 18230619–18230906**	− **1.1258168**	**0.18214965**	**4.58E−09**	
Aorta at diaphragm	cg04838249	12	34,500,640	F		chr12: 34500550–34500814	3.92396387	0.65291884	1.10E−08	
Aorta at diaphragm	cg05265042	14	102,030,999	F	DIO3	chr14: 102025989–102031567	2.89385142	0.54443147	3.31E−07	Heart failure
										Ventricular remodeling
**Aorta at diaphragm**	**cg09689342**	**16**	**5,077,985**	**F**	**NAGPA**		− **4.945524**	**0.75454621**	**6.42E−10**	
**Aorta at diaphragm**	**cg02472906**	**16**	**87,938,341**	**F**	**CA5A**		− **5.0764489**	**0.84196725**	**9.96E−09**	
Aorta at diaphragm	cg06906965	19	58,450,175	R		chr19: 58446336–58446800	− 3.9017762	0.73555419	3.48E−07	
Aorta at diaphragm	cg27614967	23	153,561,283	F		chrX: 153561035–153561361	− 5.0488276	0.87988599	4.30E−08	
**Aorta at bifurcation**	**cg24073777**	**1**	**10,832,698**	**F**	**CASZ1**		− **9.4883778**	**1.54559989**	**5.68E−09**	**Congenital heart disease**
										**Hypertension**
**Aorta at bifurcation**	**cg03158772**	**3**	**14,768,188**	**F**	**C3orf20**		− **6.4625567**	**1.0591771**	**6.89E−09**	
Aorta at bifurcation	cg24324446	3	110,363,281	F			− 4.3209801	0.79936682	2.16E−07	
Aorta at bifurcation	cg05149776	5	140,870,164	R	PCDHGA1	chr5: 140871064–140872335	− 5.8834917	1.0294066	4.81E−08	Atherosclerosis
Aorta at bifurcation	cg24588058	7	76,591,673	F		chr7: 76588998–76589608	− 4.9102623	0.94591786	5.91E−07	
Aorta at bifurcation	cg19804570	18	44,618,587	R	KATNAL2		− 5.4528539	1.01978524	2.85E−07	
Aorta at bifurcation	cg18075379	20	61,788,662	F		chr20: 61788160–61788669	− 5.1667712	0.95877471	2.34E−07	
Aorta at bifurcation	cg14798310	22	24,234,197	F	MIF-AS1	chr22: 24236257–24237539	− 4.8972641	0.88169036	1.06E−07	
**Aorta at bifurcation**	**cg27504079**	**23**	**50,653,533**	**R**	**BMP15**		− **3.9887835**	**0.65665158**	**7.91E−09**	

In yellow the significant associations with *p* < 10^–8^

At a level of significance of *p* < 10^–6^, there were 28 sites significant; one each with diameters at the aortic root, in the ascending aorta and aortic arch, two in the proximal descending aorta, and 14 and 9 at the diaphragm and bifurcation level, respectively (Table 3). Manhattan-plots as well as scatterplots illustrating the significant associations are given in Additional file 1: Figures SD2 and SD3.

Of these 28 CpG sites with a significance of *p* < 10^–6^, 19 CpG sites could be allocated to a gene (including the six with *p* value *p* < 10^–8^) (Table 3). Upon studying these genes for a possible role in MFS, four genes (FNBP1, EHBP1, SDK1, and PTPRN2) were found to be involved in cytoskeletal actin dynamics, which regulates cell-adhesion and cellular uptake or secretion [23–26], which is altered in MFS cells [27], and thought to play a role in aneurysm formation [28].

Most interestingly in relation to aortic diameters, seven genes were found to have a known role in the cardiovascular system, of which **HDAC4** is the most widely studied in cardiovascular disease.

HDAC4 is a histone deacetylase with very weak histone deacetylase activity. It actually can modulate histone methylation, which contributes to regulation of gene transcription [29]. Reviews on HDAC4 reveal functions for this protein in development of cardiac hypertrophy and remodeling [30, 31]. Apart from cardiac muscle functions, HDAC4 also plays a role in smooth muscle cell development and phenotype [32–37]. Smooth muscle cell phenotype switching is observed in the aorta of MFS mice and in human ascending aortic aneurysm tissue [38, 39].

**IGF2BP3** and **CASZ1** have key functions in cardiac development, since defects in these genes lead to congenital heart disease [40–43]. **SDK1** and CASZ1 variants are associated with hypertension [44, 45]. In addition, a SNP in **PCDHGA1** is associated with carotid artery–intima media thickness (IMT) in humans as readout of atherosclerosis [46], and **DIO3** is enhanced in cardiac tissue in heart failure and ventricular remodeling [47, 48].

For a number of these genes DNA methylation differences relate to cardiovascular disease. Hypomethylation of the HDAC4 gene promoter in genomic DNA from peripheral blood of obese adults compared to non-obese controls shows a strong correlation with reduced brachial artery flow-mediated dilation (FMD) and insulin signaling, as readout of vascular (dys)function [49]. Reduced FMD is also observed in MFS patients and correlates to aortic diameters [50, 51]. DNA methylation in placental tissue at sites encoding **PTPRN2** and CASZ1 are associated with cardiometabolic disease in adulthood [52], and DNA methylation in blood leukocytes at the location of the PTPRN2 gene is associated with future myocardial infarction [53].

### Differentially methylated positions associated with non-cardiovascular phenotypes

No significant associations at *p* < 10^–8^ or *p* < 10^–6^ were found with any of the non-cardiovascular phenotypes MFS features (Additional file 1: Figure SD4).

### Differentially methylated positions associated with aortic diameter change or aortic events

Apart from associations with aortic diameters, there were seven CpG sites with methylation levels associated (*p* < 10^–6^) with aortic diameter *change* and five CpG sites associated with the occurrence of clinical events. Details of these CpG sites are given in Table 4. Manhattan-plots and scatterplots are given as Additional file 1: Figures SD5 and SD6.

**Table 4:** CpG-sites with genome-wide significant association (*p* < 10^–6^) with change in aortic diameter or aortic events

Aortic dilatation rate	CpG	CHR	Position	Strand	Gene	UCSC_CpG_Islands_Name	*β* value	SE	*p* value	Cardiovascular function
Aortic root	cg00702593	21	42,219,853	F	DSCAM	chr21: 42218489–42219222	− 2.32E+00	4.56E−01	9.58E−07	Congenital heart disease
Proximal des aorta	cg05230977	20	62,039,853	F	KCNQ2	chr20: 62037929–62038677	2.24E+00	4.25E−01	4.24E−07	
Distal des aorta	cg17213304	5	78,364,769	R	DMGDH	chr5: 78365298–78365711	3.68E+00	7.15E−01	7.15E−07	
Aorta at bifurcation	cg26033586	3	116,163,858	R	LSAMP					Smooth muscle cell development
										Coronary artery disease
Aorta at bifurcation	cg24219974	6	14,729,722	F						
Aorta at bifurcation	cg16346212	7	83,824,255	R	SEMA3A					Congenital heart disease
										Smooth muscle cell development
Aorta at bifurcation	cg13713739	9	132,483,377	R	PRRX2	chr9:132481472–132481745				Congenital heart disease
										Malformation in aorta
*Aortic events*										
Aortic events	cg05371909	1	156,426,550	R	MEF2D	chr1: 156426549–156427362	6.797315	1.2097795	1.92E−08	Smooth muscle cell development
Aortic events	cg04316429	2	218,844,202	R	TNS1	chr2: 218843460–218843742	− 3.894949	0.7743331	4.90E−07	Cardiac valve defects
Aortic events	cg20852788	4	119,676,722	F	SEC24D		2.192283	0.4360381	4.96E−07	
Aortic events	cg17369115	6	9,476,450	F	LOC100506		− 3.724896	0.7524573	7.41E−07	
Aortic events	cg02283151	14	100,110,845	F	HHIPL1	chr14: 100111120–100111906	− 8.5573	1.7356909	8.21E−07	Myocardial infarction Hypertension
										Coronary artery disease

Within the seven CpG sites that relate to aortic diameter *change*, four genes located in or in close proximity to these CpG sites have a known cardiovascular function, namely **LSAMP**, **DSCAM**, **SEMA3A**, **PRRX2**. LSAMP has been shown to influence smooth muscle cell proliferation [54], and LSAMP variants are associated with survival in coronary artery disease patients [55]. DSCAM overexpression leads to congenital heart defects [56] and is probably responsible for the congenital heart defects observed in Down syndrome patients [57]. Moreover, SEMA3A and PRRX2 mutations also lead to congenital heart defects [58, 59]. Next to its function in the heart, both proteins also influence smooth muscle development. SEMA3A is mechanosensitive and influences smooth muscle cell recruitment for vascular maturation [60], while deficiency of PRRX2 leads to malformations of the aorta in mice [61].

Within the five CpG sites that relate to aortic events, three genes located in or in close proximity to these CpG sites have a known cardiovascular function, namely **MEF2D**, **TNS1** and **HHIPL1**. Of these genes MEF2D is most widely studied in cardiovascular disease, since the family of MEF2 transcription factors is regulators of skeletal, cardiac and smooth muscle cell development [62]. TNS1 connects extracellular matrix to the cytoskeletal interior [63]. Alterations in TNS1 lead to cardiac valve defects causing mitral valve prolapse [64, 65], as is also observed in MFS patients [66]. Finally, variants in HHIPL1 are associated with myocardial infarction, increased blood pressure and coronary artery disease, probably in part, because smooth muscle cell-derived HHIPL1 enhances atherosclerosis as reported in two hyperlipidemic mouse models [67, 68]. The methylation state of these CpG-sites could influence gene expression levels and may represent functional involvement of the genes they represent in the methylation profile of the white blood cell is similar in the cardiovascular tissue.

**Fig. 2:**
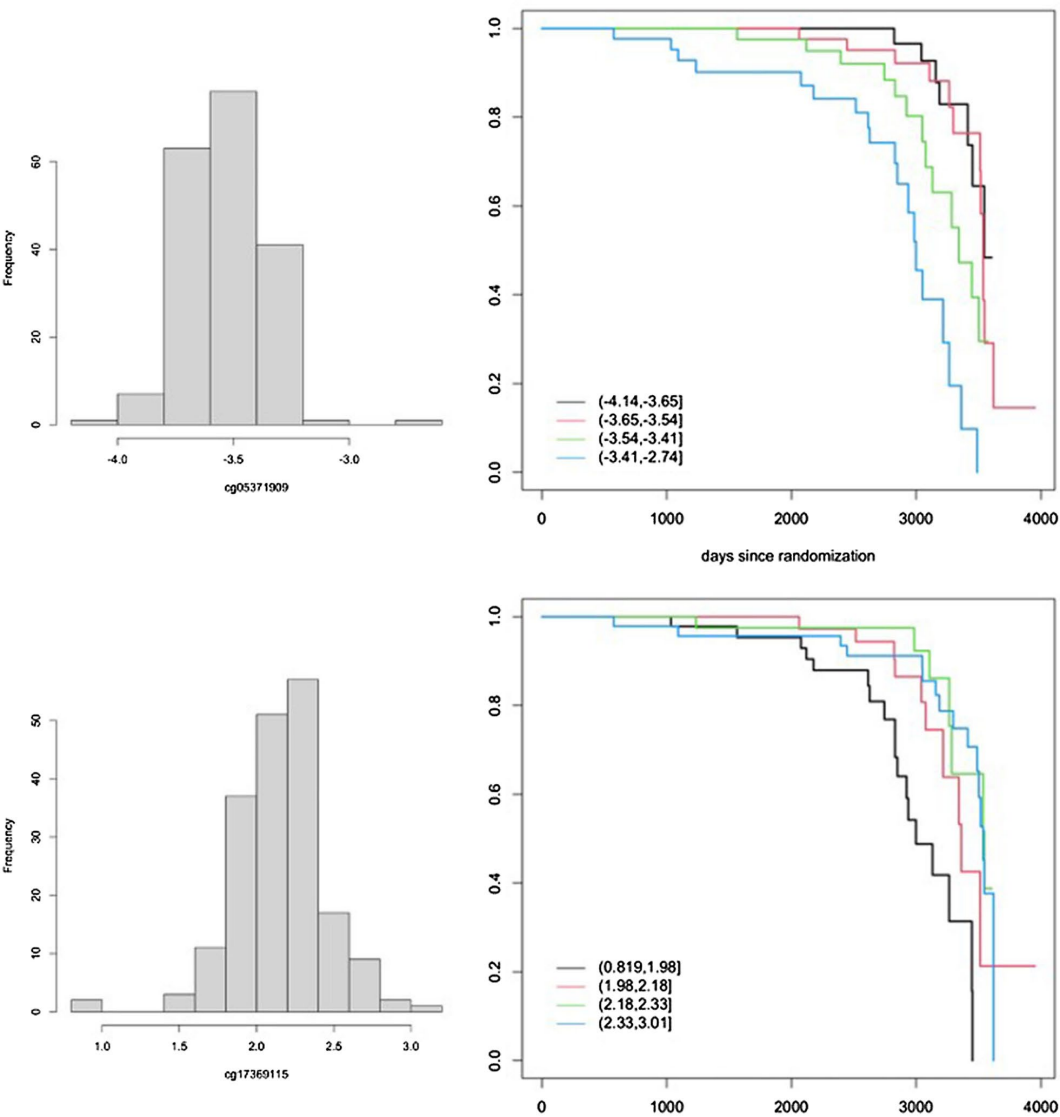
Kaplan–Meier curves of time to first clinical event in subgroups defined by quartiles of the distribution of methylation levels at 2 CpG sites, representing hypermethylation of cg05371909 site (MEF2D) or hypomethylation of the cg17369115 site (LOC100506) is associated with events

Association of time to first clinical event with methylation levels at cg05371909 (MEF2D) and cg17369115 is illustrated in Fig. 2 with Kaplan–Meier curves defined by the quartiles of the methylation distributions. Here we show examples of a hyper- and hypomethylation associated with CpG site with events.

### Differentially methylated regions

**Fig. 3:**
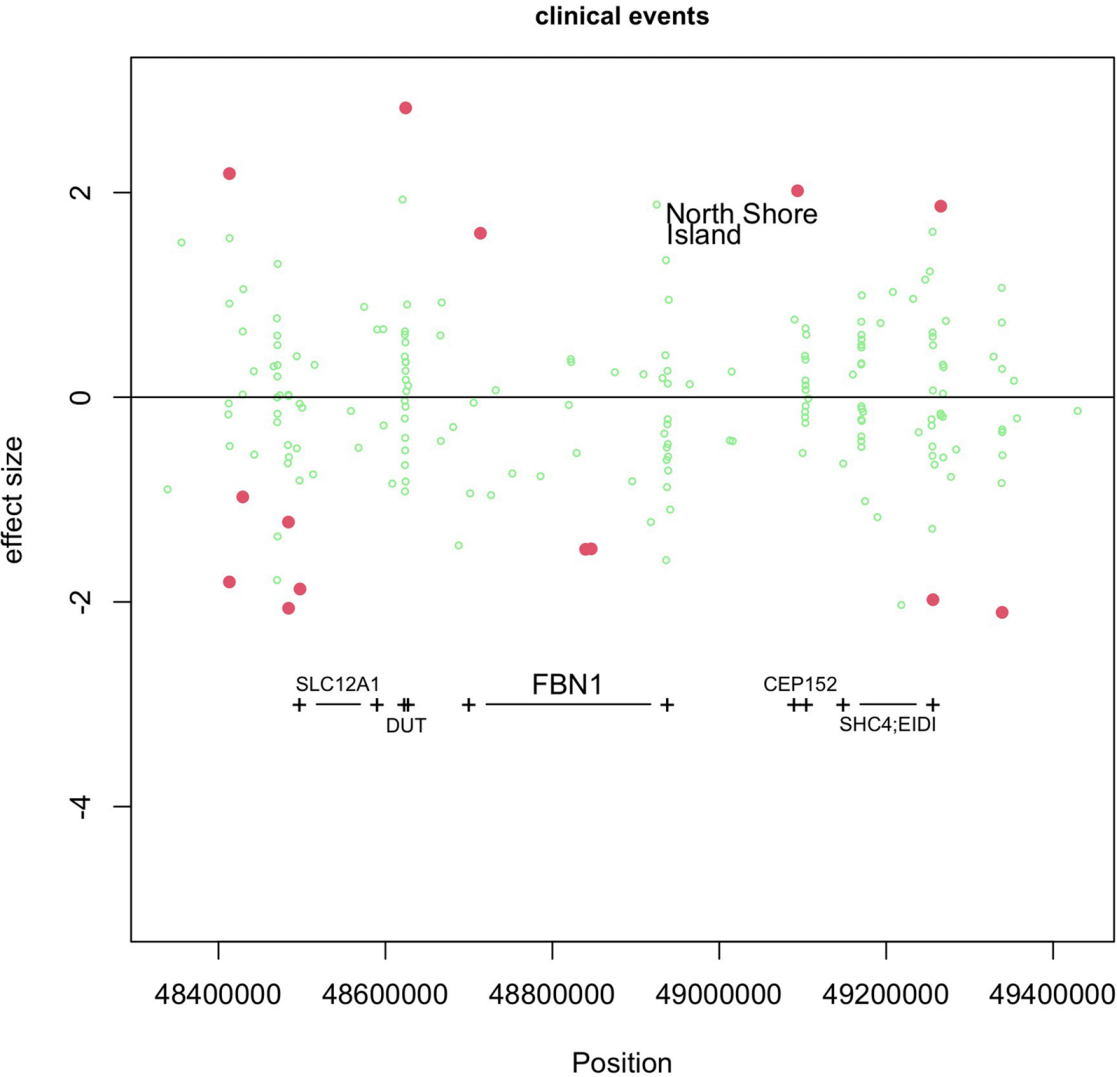
Log Hazard Ratio of M-values at CpG sites in and close to the FBN1 gene on chromosome 15

Smoothed standardized regression weights of baseline phenotypes on M-values and of aortic diameter *change* and clinical *events* were done on M-values. We zoomed in on the association of the incidence of clinical events with M-values of CpG-sites in and close to the FBN1-gene in Fig. 3, but no significant DMRs were observed for this region.

In total 95 putative DMRs were found with baseline phenotypes and 34 with diameter *change* and clinical *events* of which 15 + 9 and 3 + 10, respectively, were located on the X- and Y-chromosomes. Of the 71 and 21 other DMRs, 45 and 17 DMRs were located on chromosomes 18 to 22. Because these chromosomes are smaller with fewer CpGs, we think that DMRs on these chromosomes are more susceptible for the choice of the span-width of the loess-smoother and are therefore less reliable. Numbers of DMRs on other chromosomes were mostly 0 or 1, with the exception of chromosome 5, where 14 DMRs for baseline phenotypes are found.

CpGs in the 95 and 34 DMRs involved in baseline phenotypes and aortic diameter *change* and clinical *events* were located in or in close proximity of 63 and 49 genes, respectively, with 25 genes overlapping in both sets of DMRs (Additional file 1: Table sT1). Among the genes behind the DMRs there were two well-known cardiovascular transcription factors, namely Notch1 and Tbx1, which are also involved in cardiovascular development [69, 70]. When combining these two gene lists, gene-set enrichment with gene ontology and genemania showed that genes in the ‘homophilic cell adhesion via plasma membrane adhesion molecules’ as biological process and ‘calcium dependent cell-cell adhesion’ in particular, were enriched (13/18 in the lists versus 0.36/0.42 as expected: *p* values = 4.16e−16 and 5.57e−26, FDR  = 6.61e−12 and 8.84e−22, respectively).

It becomes evident that the largest group of genes represents a protocadherin gene cluster on chromosome 5. These genes have an immunoglobulin-like organization; the genomic DNA sequences encoding their ectodomains are not interrupted by an intron, thus these genes have unusually large exons, next to three constant exons that they all have in common [71]. Protocadherins are involved in cell adhesion and have been shown to regulate tissue development, and many of them are expressed in the heart [72]. Among the protocadherin genes are the previously mentioned PCDHGA1, but also PCDHGA4, of which mutations lead to atrial septal defects [43], PCDHA9 mutations playing a role in valvular defects [73], and altered PCDHGA3 gene expression is strongly associated with reduced stroke volume and ventricular dysfunction [74].

By identification of DMRs, the large number of genes in proximity of these regions allow for gene-set enrichment analysis to unravel pathways that are overrepresented. Here, the protocadherin gene cluster largely determines the identified pathway of altered cell adhesion.

## Discussion

### Key findings

Our EWAS study identifies genetic loci potentially influencing characteristic features of patients with MFS. Among the identified differentially methylated genetic loci in or near candidate genes, several of these genes have been implicated in other cardiovascular phenotypes, potentially also affecting cardiac and/or aortic phenotype in MFS. Twenty-eight DMPs that were identified associated with baseline aortic diameters, seven DMPs with aortic diameter *change*, and five DMPs with clinical *events*. No DMPs were significantly associated with the other 21 MFS features that were analyzed. As for the DMRs, the large number of DMRs and related genes allowed gene-set enrichment analysis, which revealed the process of cell adhesion, that could for a large part be traced back to a cluster of protocadherins on chromosome 5. The relevance of these protocadherins in MFS still has to be determined. No methylation loci were identified in the FBN1 gene vicinity to associate with MFS phenotype.

### Discussion of the key findings

Based on findings from others [8, 14, 17], it was anticipated we would identify differently methylated loci surrounding the FBN1 gene that may influence FBN1 expression. However, we were not able to strengthen these results with our own findings.

Regarding the DMPs, it was striking that a number of the underlying genes had a known cardiovascular function, since these DMPs are identified in DNA of peripheral whole-blood samples, thus DNA of white blood cells. Of these genes, the cardiovascular function often represented cardiovascular development. This suggests activation of tissue repair processes, since wound healing is known to activate the fetal gene program [75]. If the DMPs in the white blood cells are similarly affected in the cardiovascular tissues remains to be determined. Only then, these genes may be functionally involved to serve as potential target for treatment to halt aortic aneurysm growth

Even though we detected only a small number of statistically significant differentially methylated CpG sites in DNA of whole-blood samples that are associated with aortic characteristics, these DMPs may be followed up for validation to potentially serve as biomarkers for aortic disease severity in patients with MFS. Even without knowing the function of a particular DMP, using the level of hyper- or hypomethylation showed predictive value for occurrence of clinical events. Interestingly, DNA methylation biomarkers have become a major area of research as potential alternative diagnostic method for various forms of cancer [15–17]. For example, in lung cancer it detects the early stage of disease [76]. Another study conducted in cancer patients described that DNA methylation data could improve early detection beyond known risk factors [77]. Identified DNA methylation markers may not only constitute a precision medicine tool, but may also help elucidate novel mechanisms of treatment. Both, to detect early onset of aortic disease and to unravel novel treatment mechanisms, DNA methylation may provide insight in MFS.

While we did not observe significant DMPs related to other MFS characteristics besides aortic characteristics, among the genes behind the DMPs that were significantly associated with aortic diameters, some of these genes have known functions representing other MFS characteristics. According to The Human Gene Database ‘GeneCards®,’ seven genes were identified (SAMD4A, EHBP1, IGF2BP3, PTPRN2, PIP4K2A, SLC2A11, and BMP15) that are involved in glucose transport/insulin signaling, which regulates tissue growth and cellular survival. Moreover, indicated in the same database, of these seven genes, variants in four of them (SAMD4A, IGF2BP3, PIP4K2A, and BMP15) and additionally HDAC4 have been associated with abnormal body height as is often observed in patients with FBN1 mutations (tall MFS patients or short stature acromelic dysplasia patients [78, 79]. Disturbed insulin/Akt signaling may thus be a determinant for the excessive growth in MFS patients, which deserves further investigation. Interestingly, also SEMA3A mutations may cause short stature, as can be observed in FBN1 mutation patients with acromelic dysplasias [58]. Moreover, the two genes FAM50B and NAGPA are associated with adolescent idiopathic scoliosis (GeneCards®), which is a common feature in MFS patients [78, 80].

In conclusion, our EWAS study in MFS patients provides novel leads that are worth looking into in future MFS research. Furthermore, it would be interesting to follow-up on the use of methylation status as biomarker assay for aorta disease severity or genes and pathways involved in the pathological processes in MFS.

## Limitations

A potential limitation is the use of whole-blood samples. As DNA methylation is tissue specific, we ideally would have analyzed connective tissue, such as aortic or skin tissue. However, sampling of aortic tissue in epidemiological studies is not realistic, while the use of DNA methylation from skin tissue would be of interest for comparison with our blood-derived results. Nevertheless, peripheral blood is easy to access and thought to represent a signature that is concordant with other tissue types [81]. Although research on the extrapolation of peripheral blood to cardiac tissue DNA methylation patterns is scarce, good results between cardiovascular biopsies and peripheral blood samples on DNA methylation were published previously in heart failure patients [82]. Nevertheless, it would still be necessary to investigate the reproducibility between aortic tissue and peripheral blood DNA methylation before any translational outlook could be accomplished. Moreover, epigenetic biomarkers derived from routine blood samples are more convenient for clinical practice. Another limitation is that we only have blood samples taken at baseline. Longitudinal studies assessing repeated measurement of DNA methylation would be needed to assess whether the epigenetic changes identify severity of aortic pathology.

## Conclusion

In summary, this first methylation study in MFS patients has identified a small number of differentially methylated loci in the DNA of whole-blood samples that are significantly associated with aortic diameters, aortic diameter *change* and aortic *events*. Of the underlying genes, 35% of these genes are associated with cardiovascular disease, and are thus of interest to evaluate further. Investigating methylation regions, revealed an interesting gene cluster of protocadherins on chromosome 5, which could be functionally involved in MFS by altered cell adhesion.

Further studies are needed to confirm and extend these findings, including evaluation of the functional relevance of the loci and replication in other MFS populations. Our findings add to the slowly growing literature on the epigenetic architecture of MFS patients.

## Supplementary Information

**Additional file 1**. Supplemental material.

## Data Availability

The datasets analyzed during the current study will become available from the corresponding author on reasonable non-commercial request.

## Abbreviations

DMP: Differentially methylated positions
DMR: Differentially methylated regions
FBN1: Fibrillin-1
MFS: Marfan syndrome
