# Supplementary material for: Genome-wide methylation patterns in Marfan syndrome
Source: Clin Epigenetics. 2021 Dec 11;13:217. doi: 10.1186/s13148-021-01204-4 (PMC8665617; doi:10.1186/s13148-021-01204-4)
Supplement: Supplementary file 1 — Additional file 1. Supplemental material. [file 13148_2021_1204_MOESM1_ESM.docx]

**SUPPLEMENTAL MATERIAL**

**Figure SA1.**

**
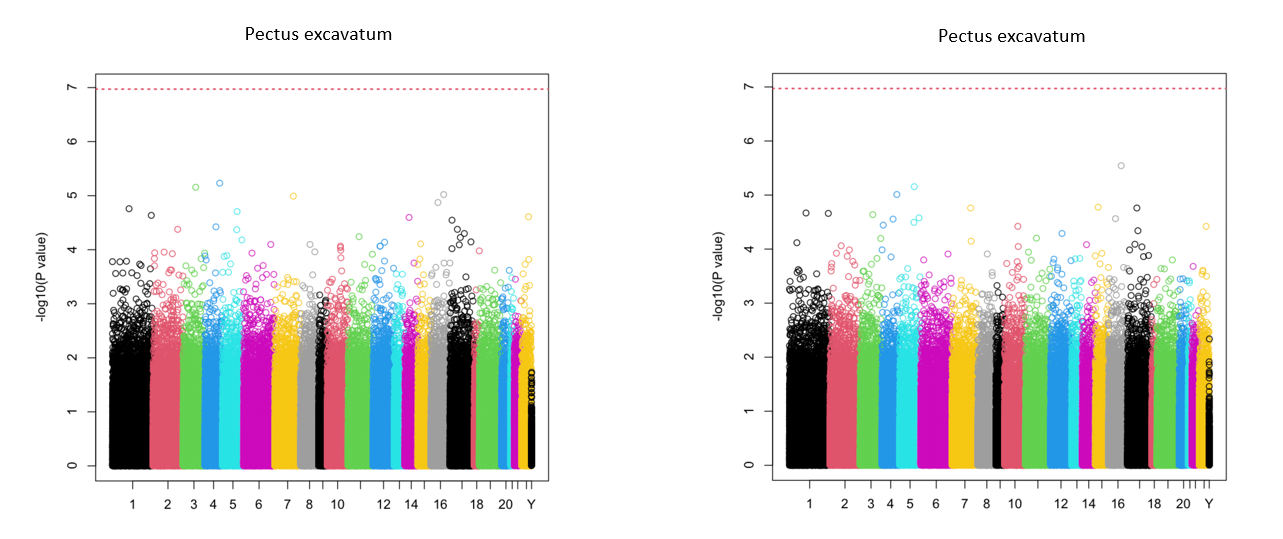

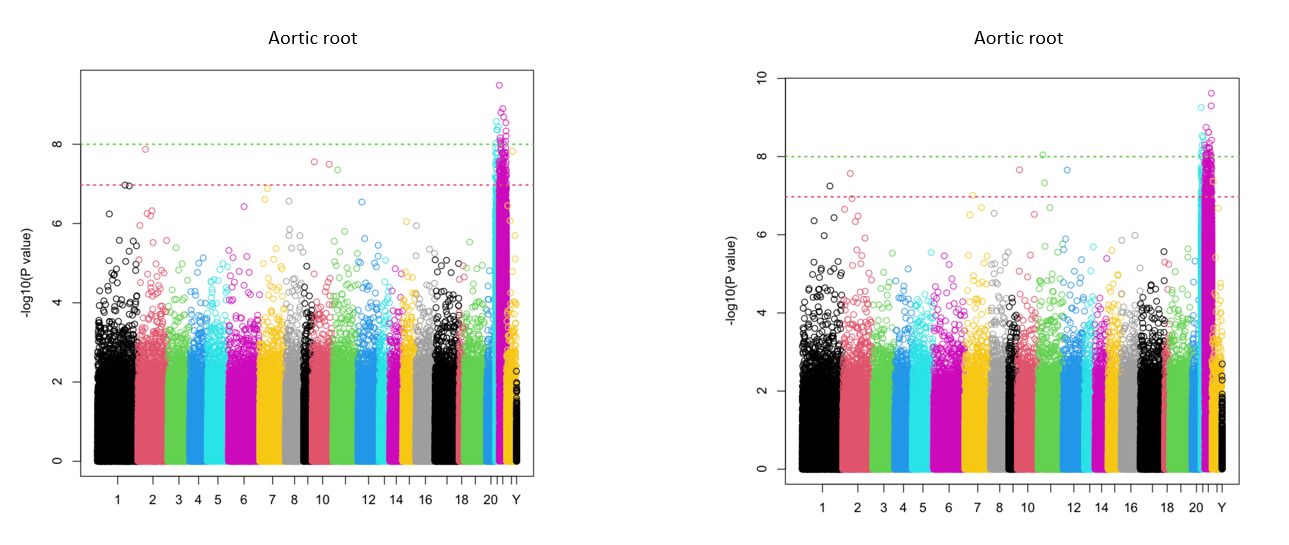

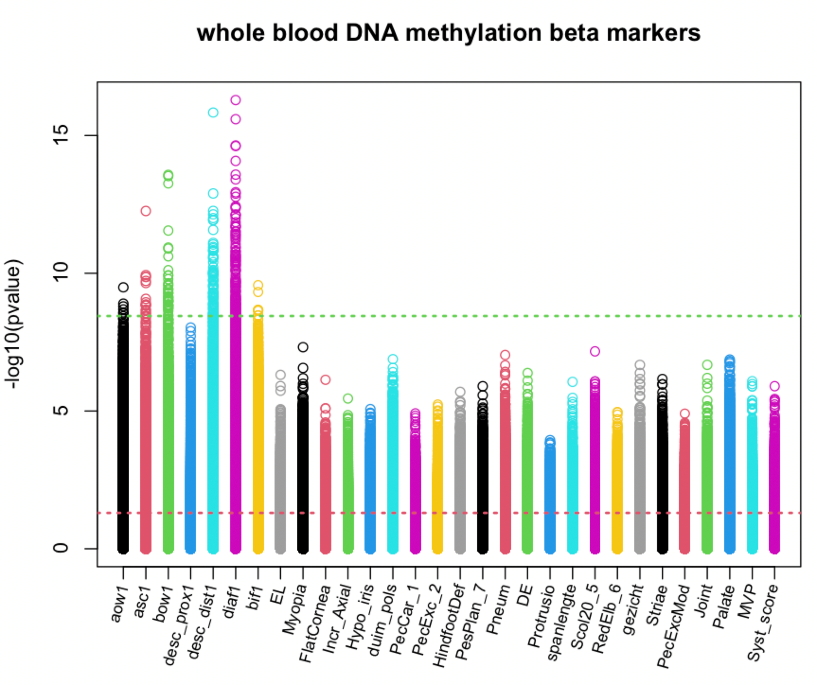
 With
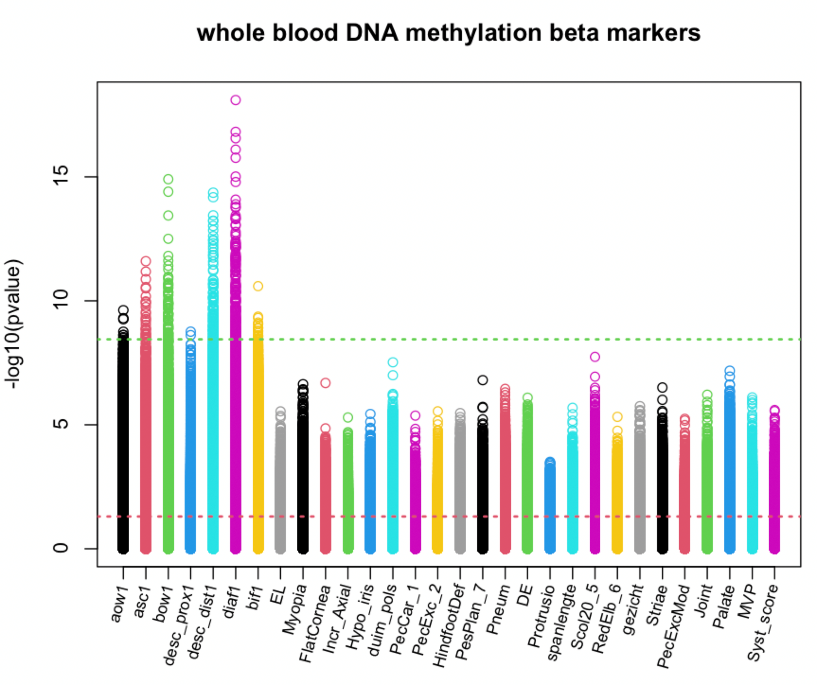
imputed data Without imputed data**

**Figure SB1.** Histograms of the baseline aortic diameters (mm).


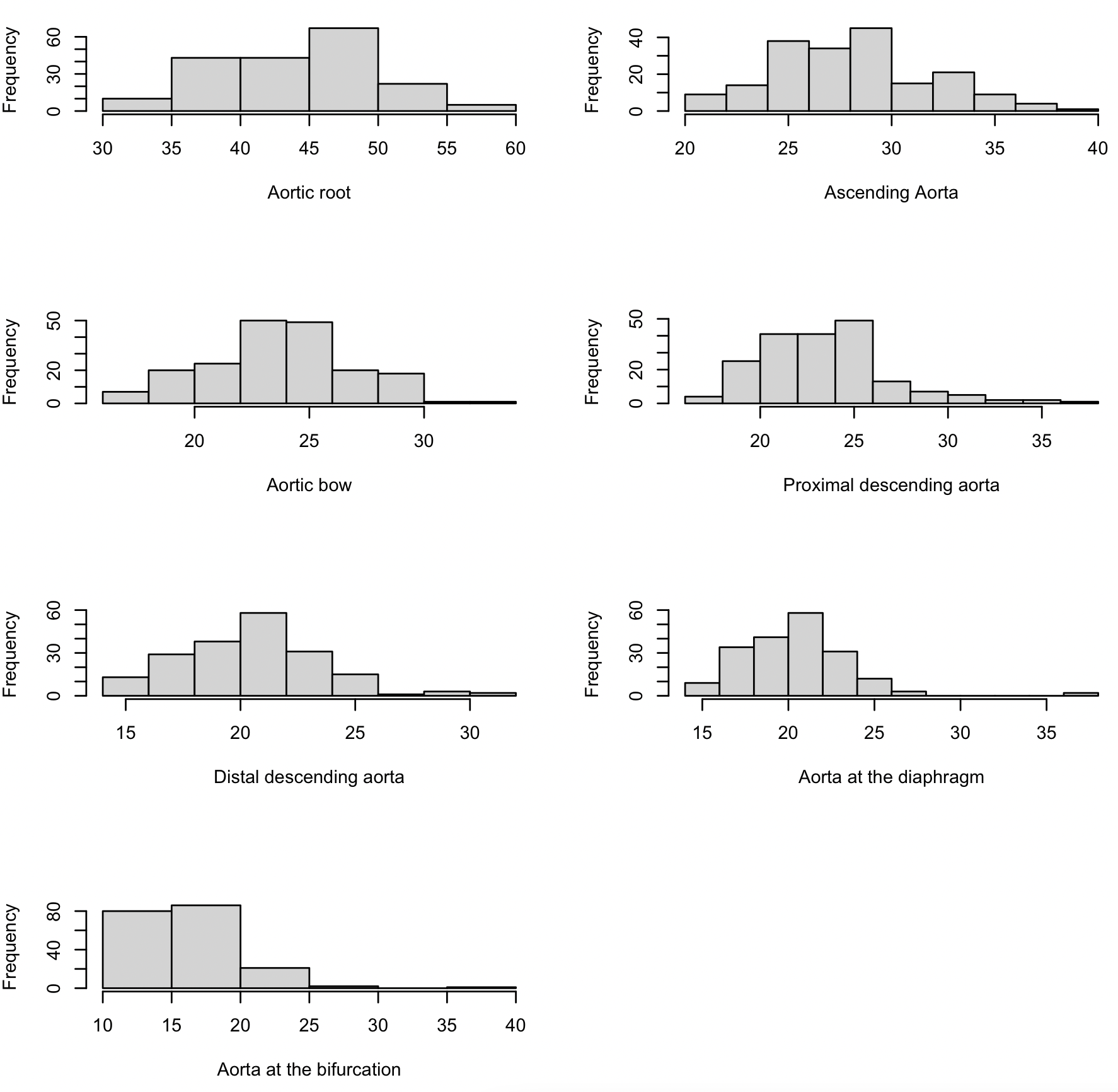


**Figure SB2.** Histograms of the *change* of the aortic diameters (mm/3-year) during the 3-year follow-up of the clinical trial.


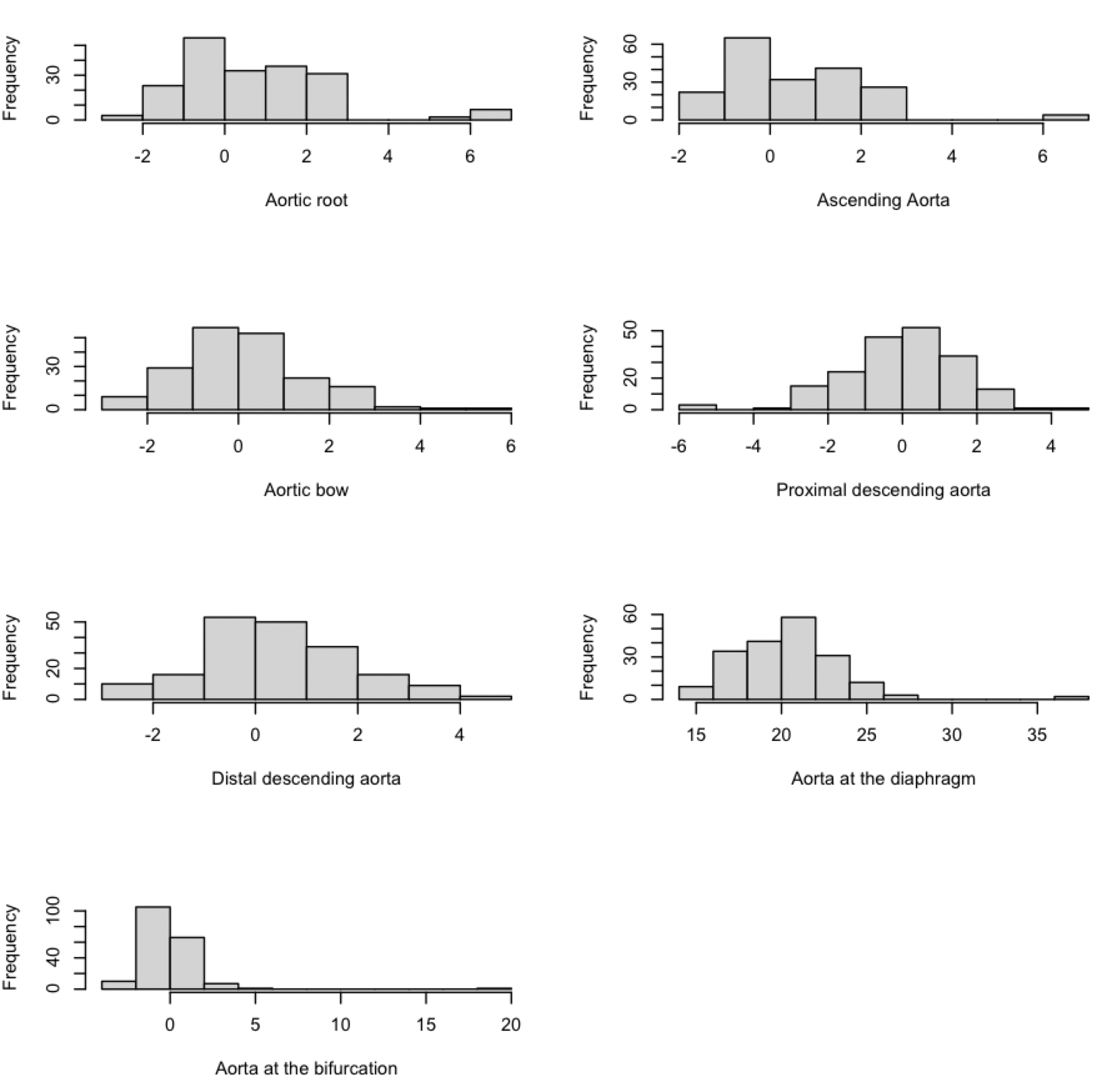


**Figure SC1.** Median log2 methylated- and unmethylated-intensity values per patient. The dotted line defines the quality-threshold of 21 for the sum of both intensities (default choice of the minfi-package).


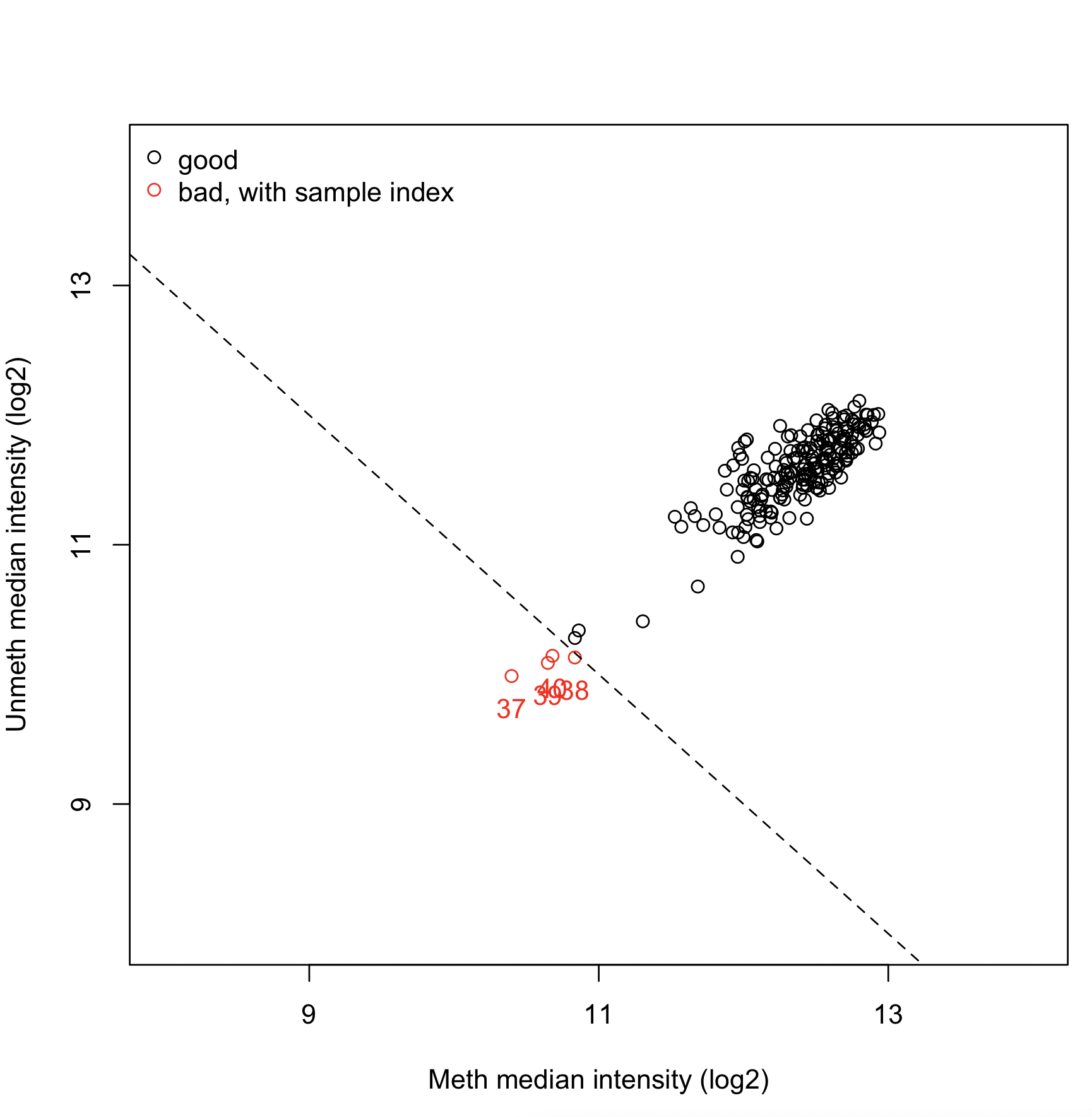


**Figure SC2.** Scree- and scatterplots of principal components analysis (A.) and illustrating association of sex (B.), age (C.) and batch number (D.) with the first 9 M-values principal components.

**A.**


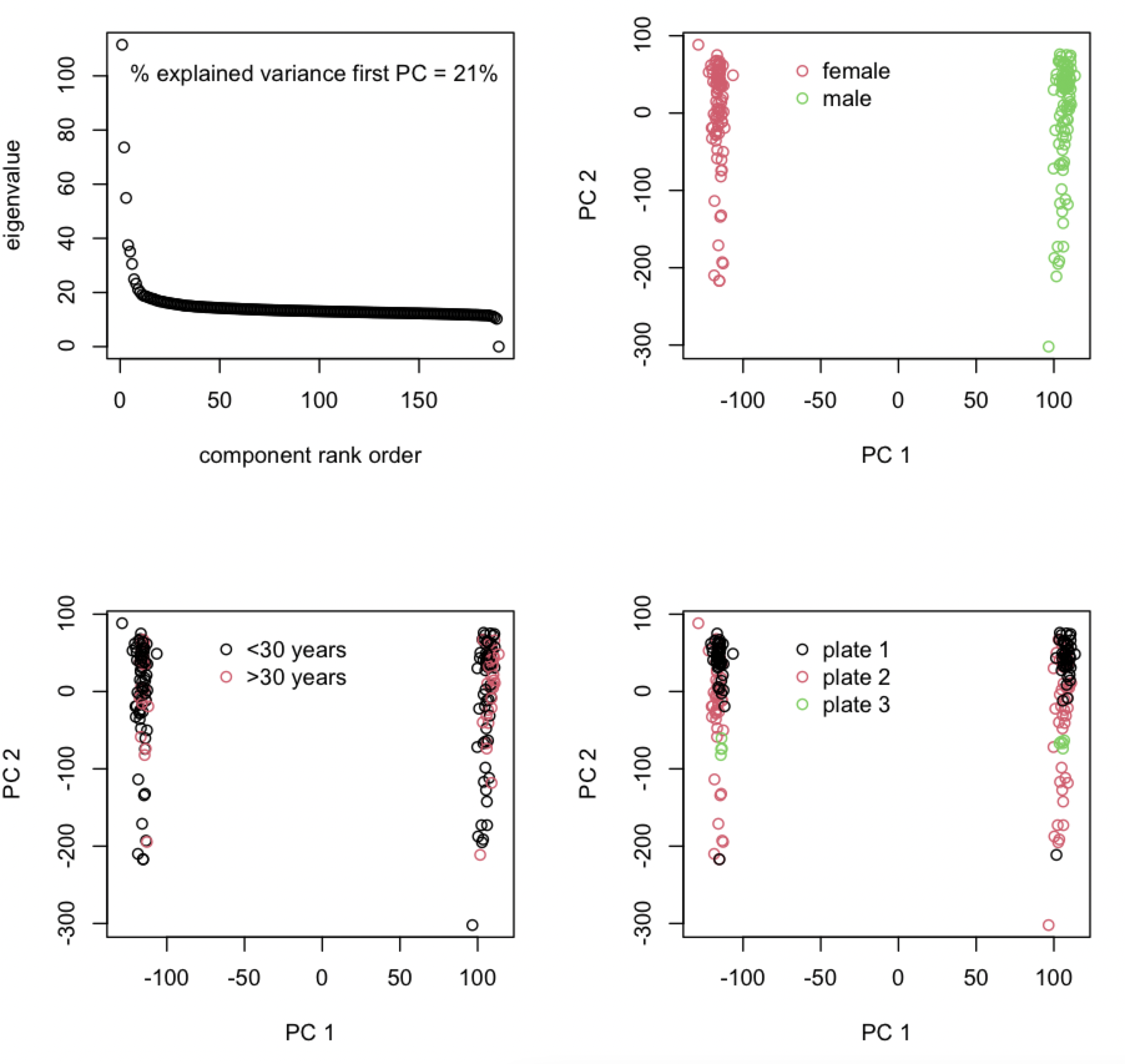


**B.**


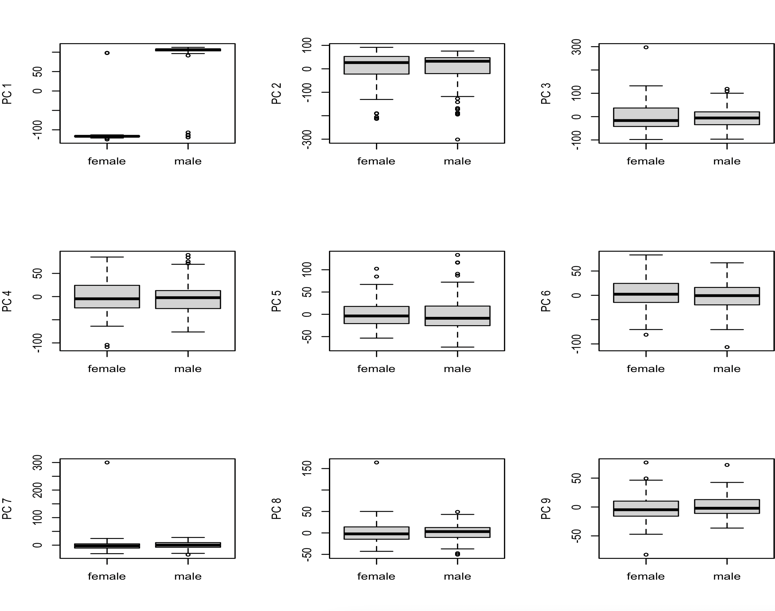


**C.**

**
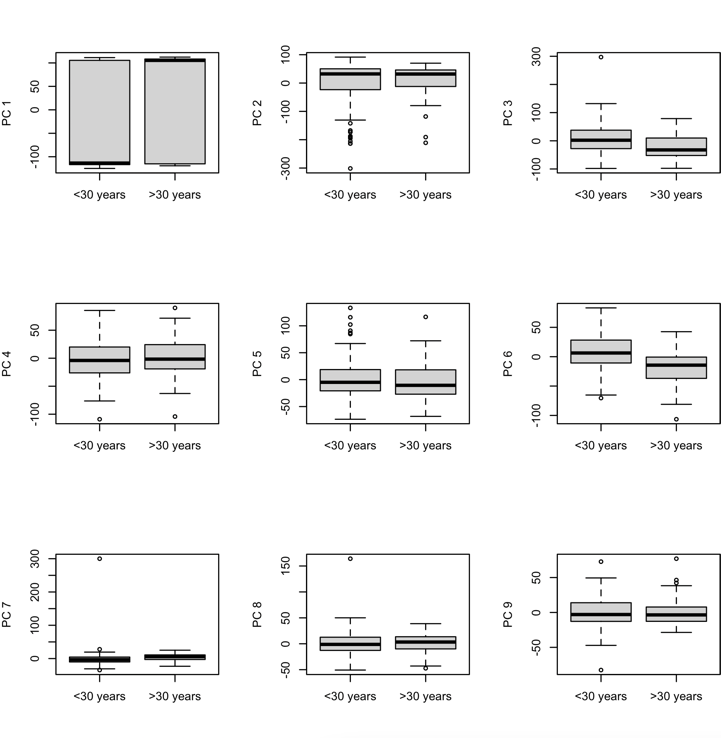
**

**D.**

**
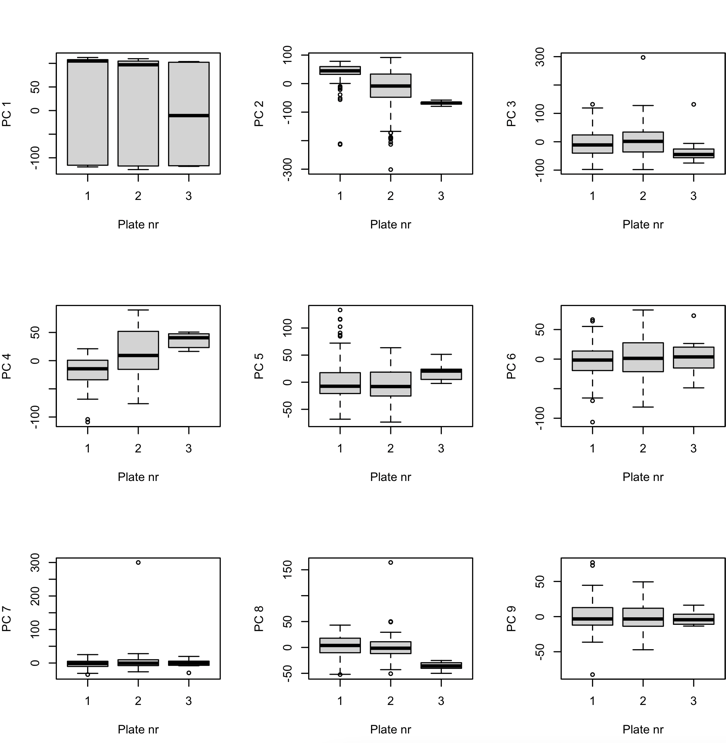
**

**Figure SD1.** Kaplan-Meier curve of the combined incidence of events (death, rupture, dissection, aortic surgery) until 2018.


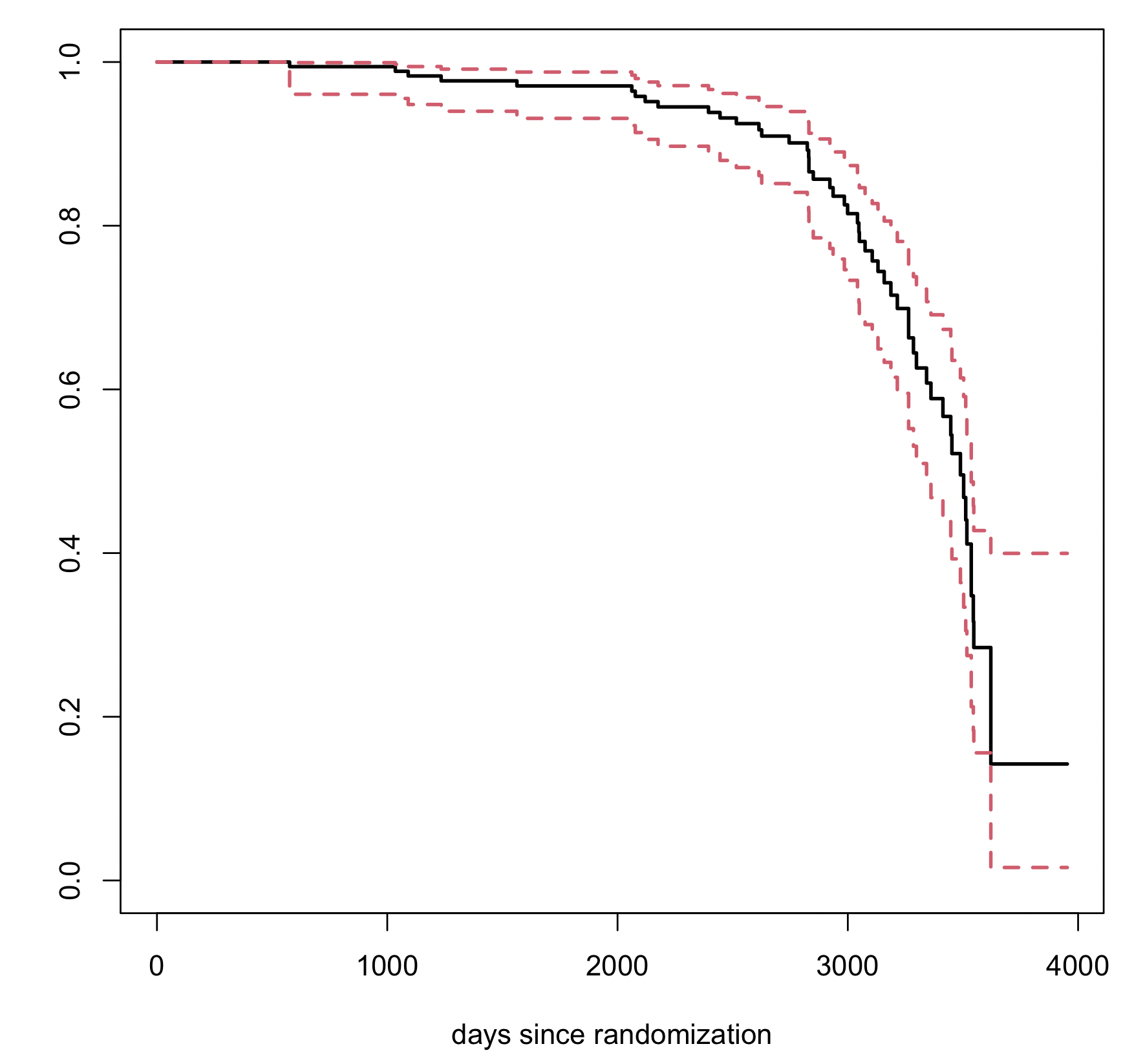


**Figure SD2.** Manhattan plots of -log^10^(P-values) of the association of baseline aorta diameters with genome wide methylation levels corrected for age, sex, BSA and estimated cell-type distribution variables.


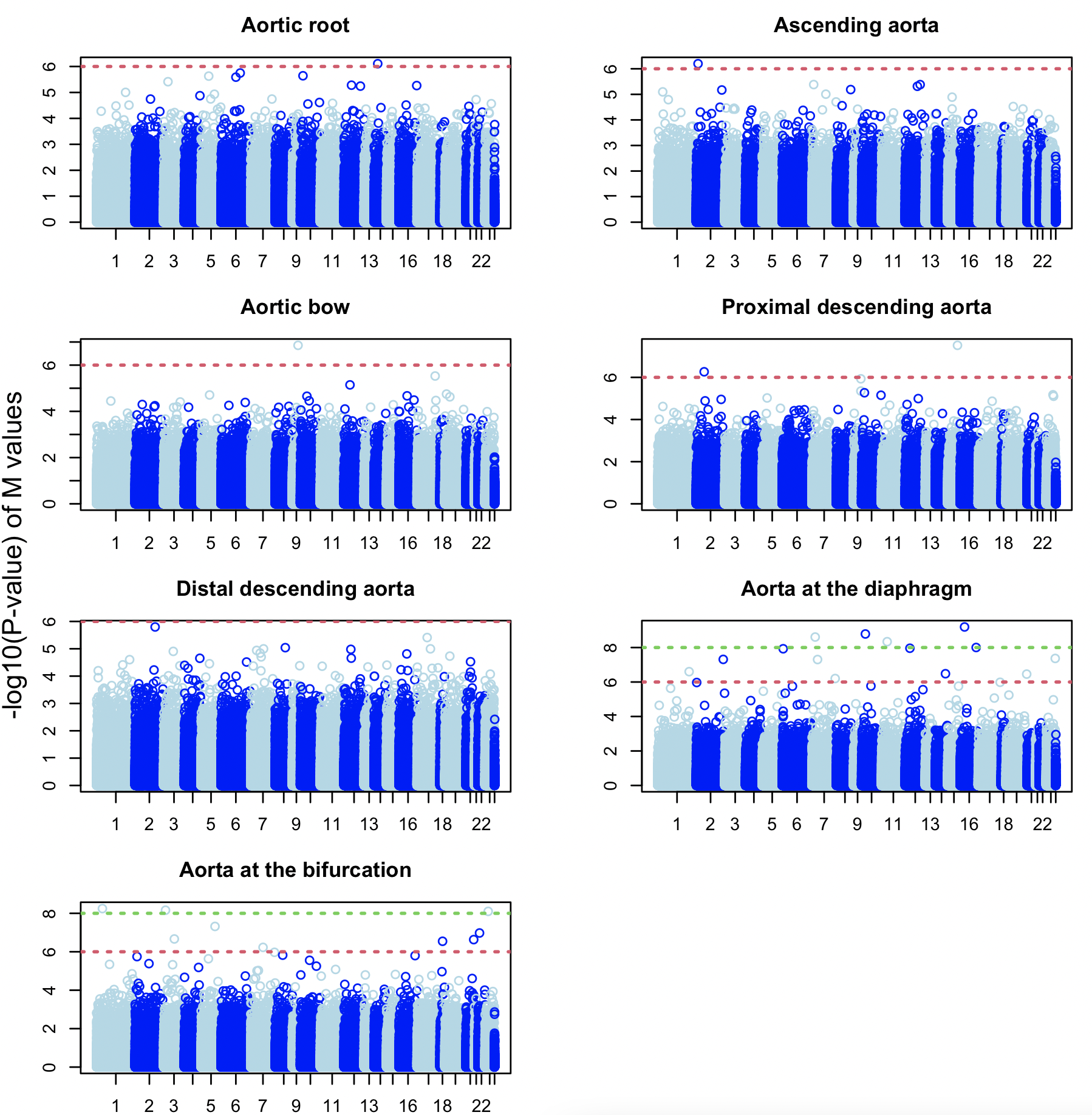


**Figure SD3.** Scatterplots illustrating association of baseline aorta diameters with methylation levels at 28 CpG sites with p-values < 10^-6^.

**A.** Aortic root, Ascending aorta, Aortic Bow, and Proximal Descending aorta.


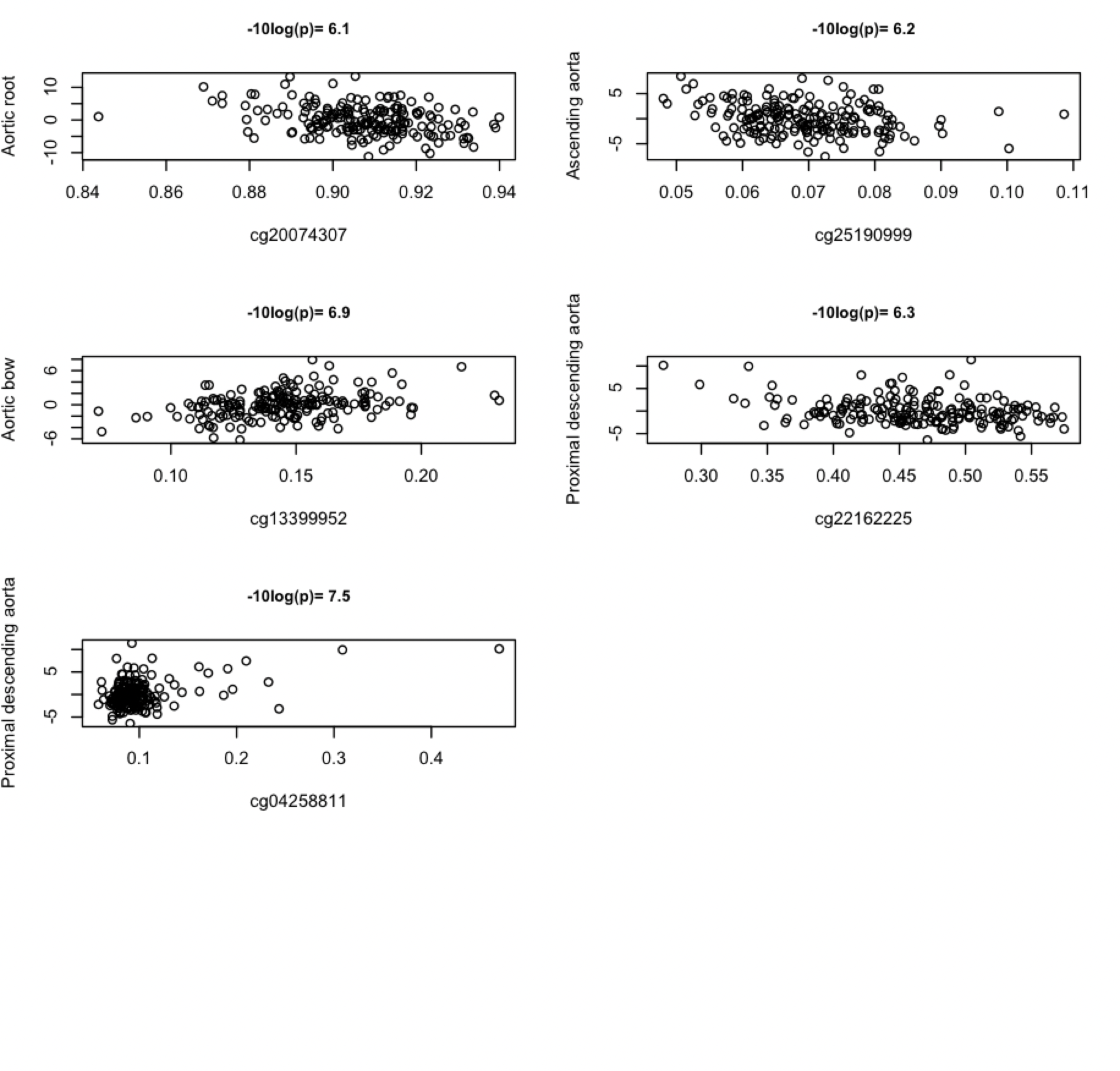


**B.** Aorta at the diaphragm.


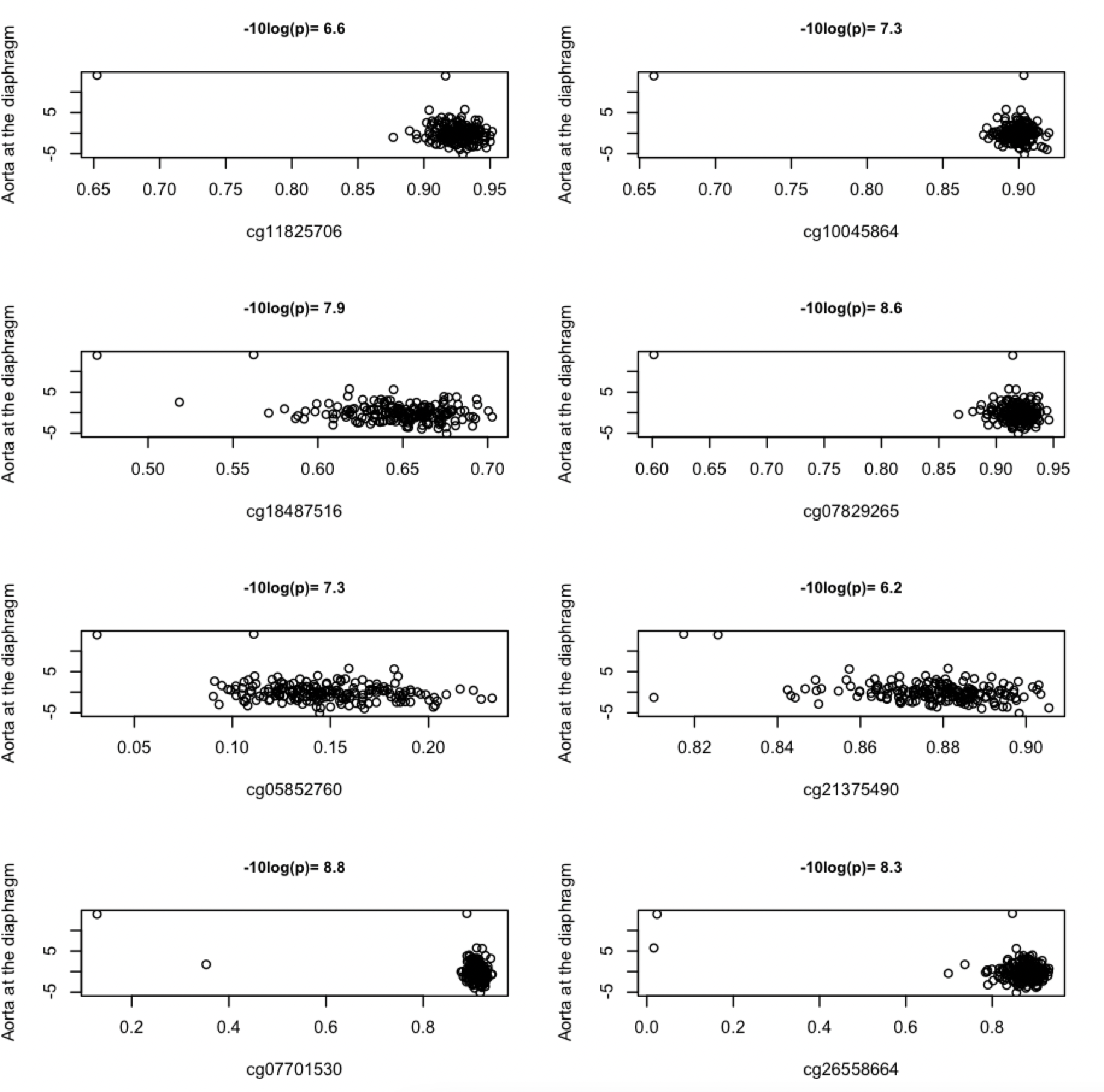


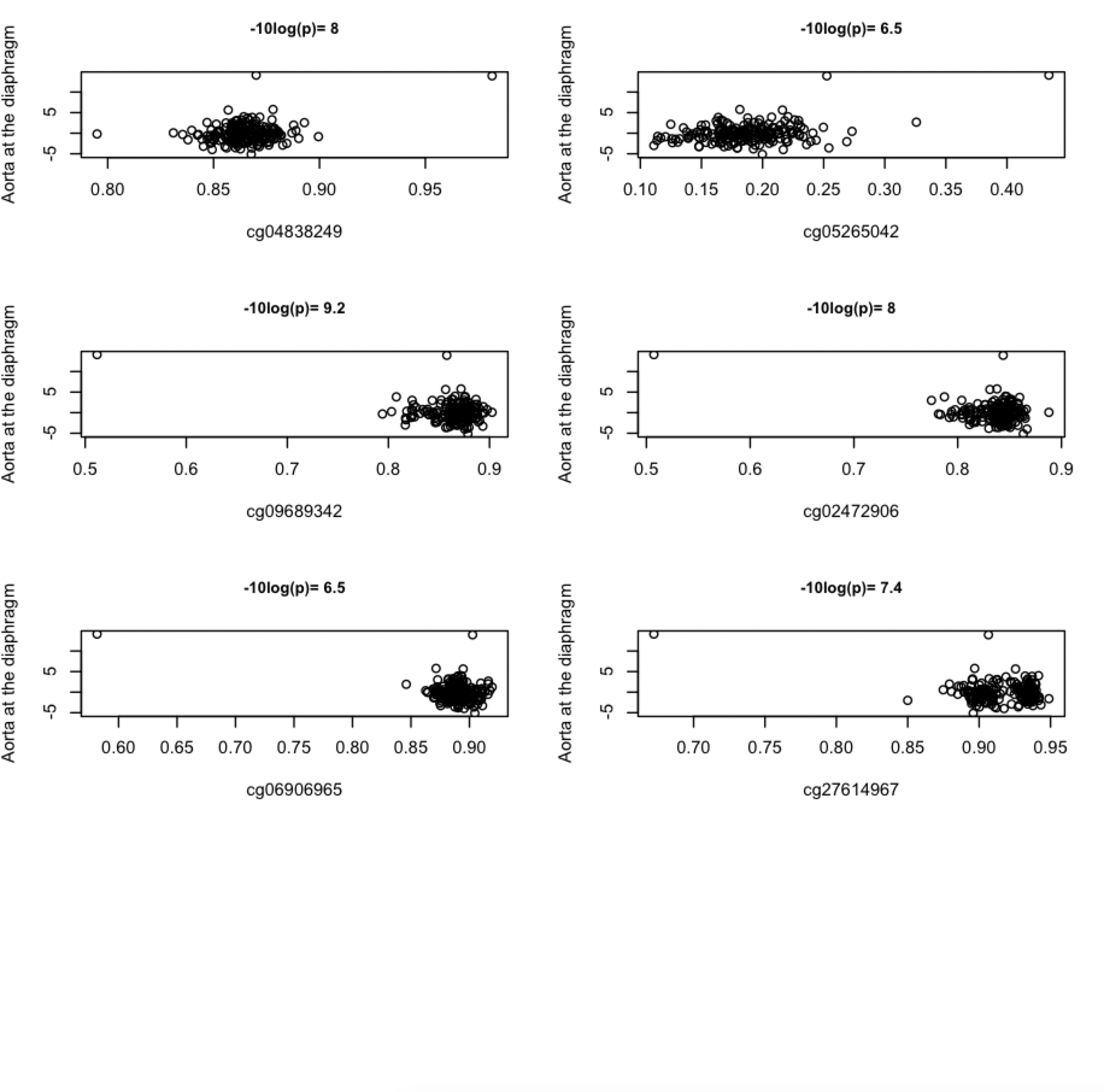


**C.** Aorta at the bifurcation.


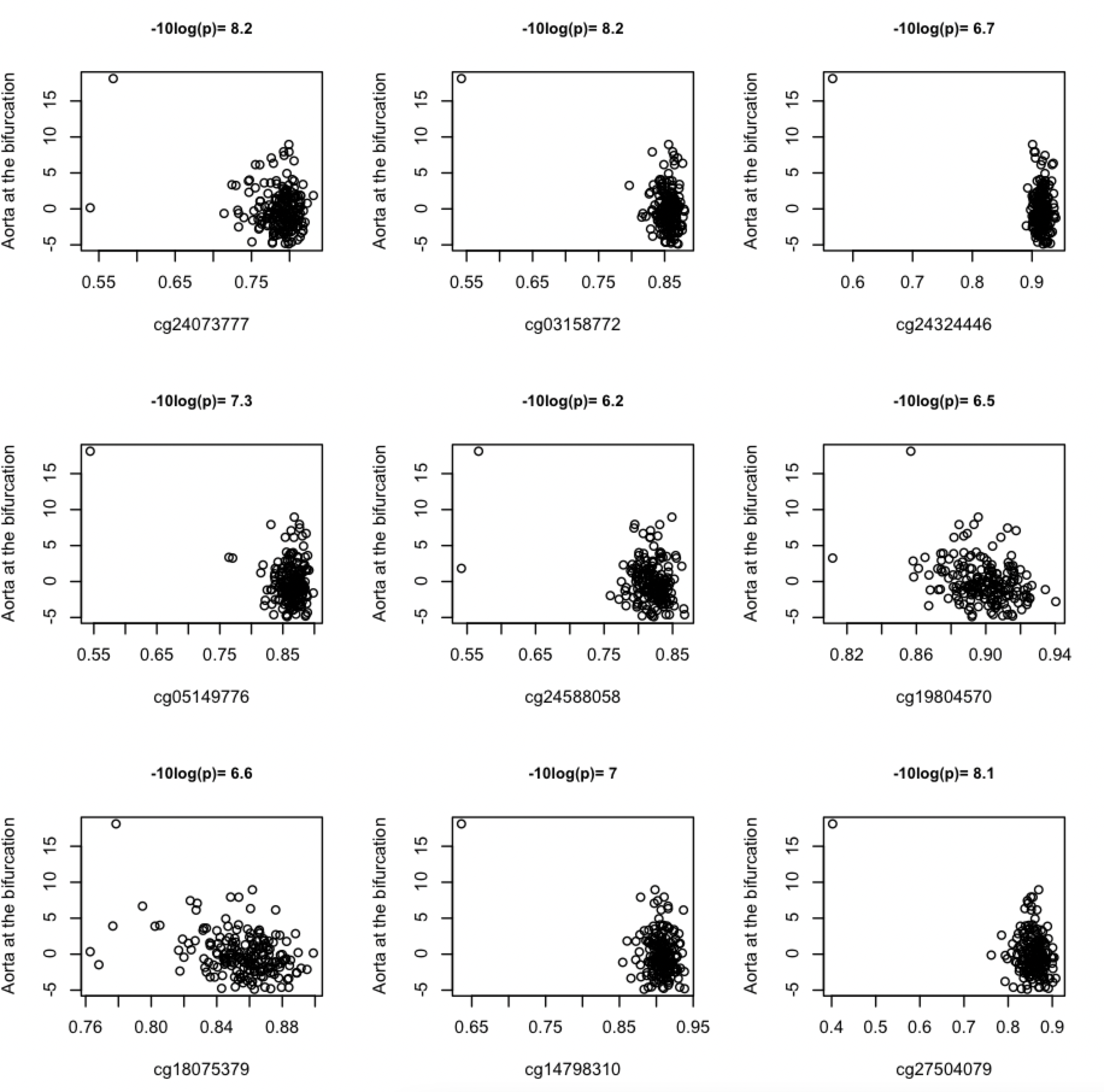


**Figure SD4.** Manhattan plots of -log^10^(P-values) of the association of baseline non cardiovascular Marfan features with genome wide methylation levels corrected for age, sex, BSA and estimated cell-type distribution variables.


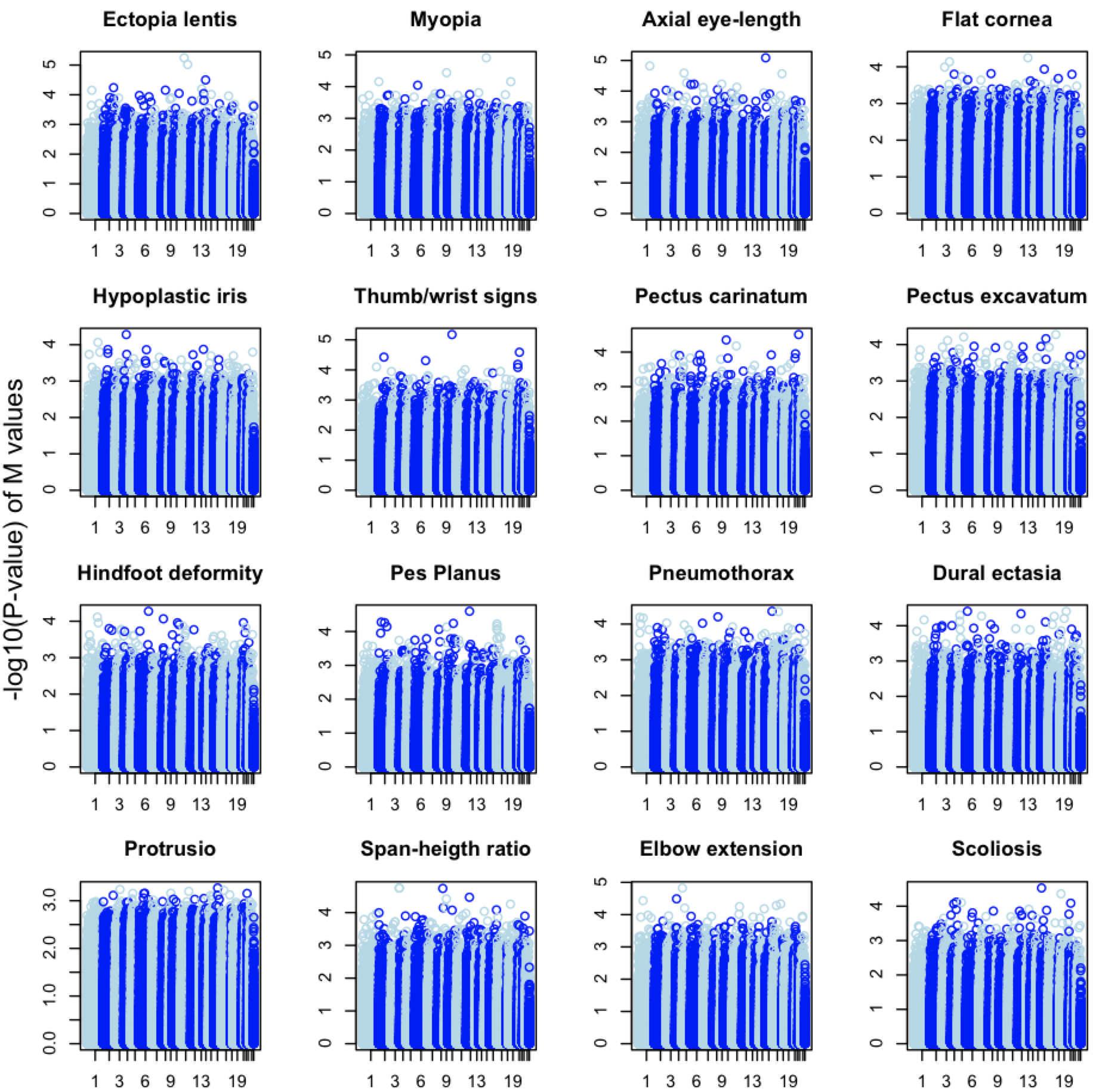


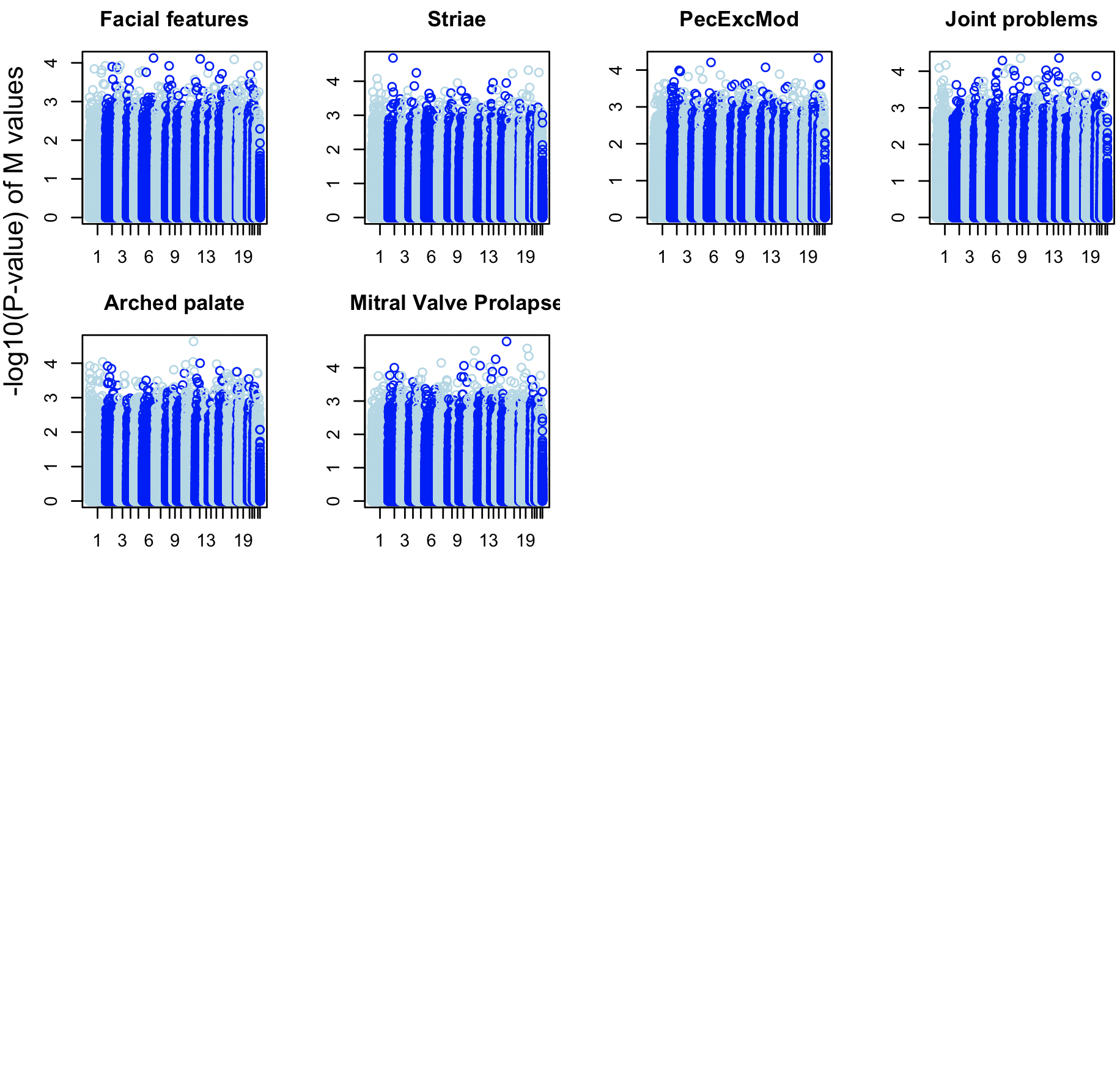


**Figure SD5.** Manhattan plots of -log^10^(P-values) of the association of change in aortic diameter and the occurrence of events with genome wide methylation levels corrected for age, sex, BSA and estimated cell-type distribution variables.


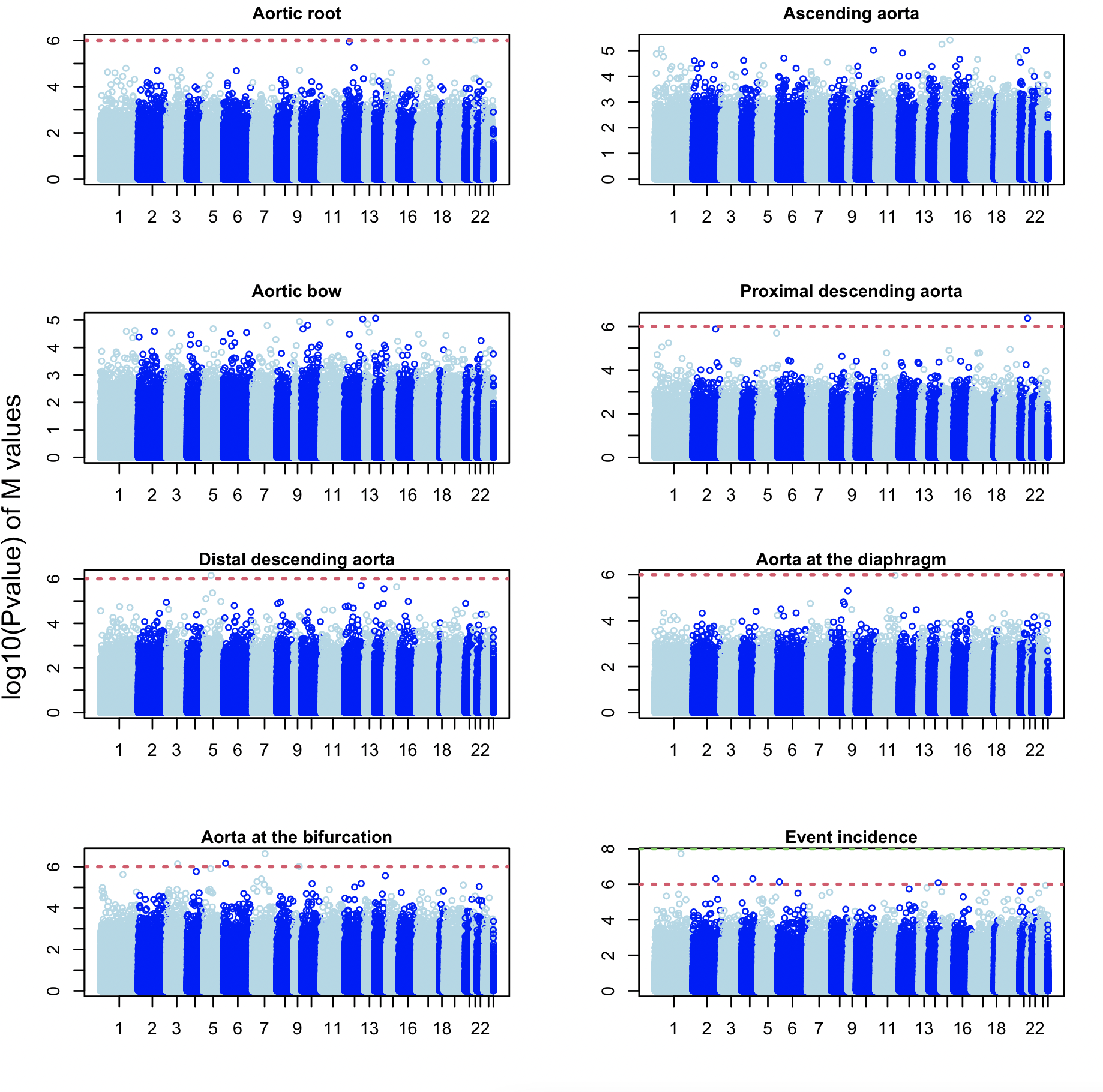


**Figure SD6.** Scatterplot illustrating association of change in aortic diameter with methylation levels at different CpG sites with p-values < 10^-6^.

**A.** Aortic root, Proximal- and Distal Descending aorta.


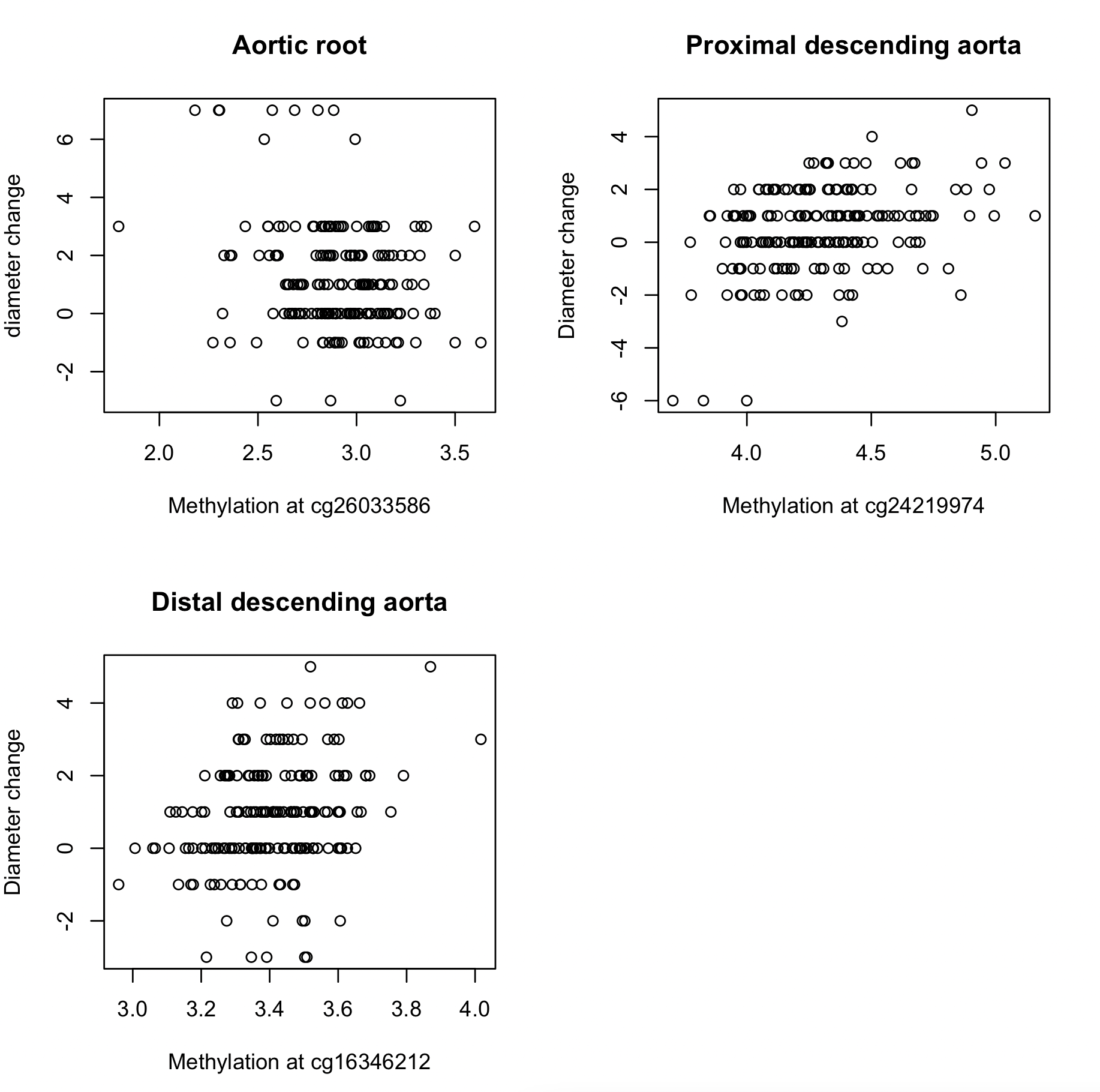


**B.** Aorta at the bifurcation.


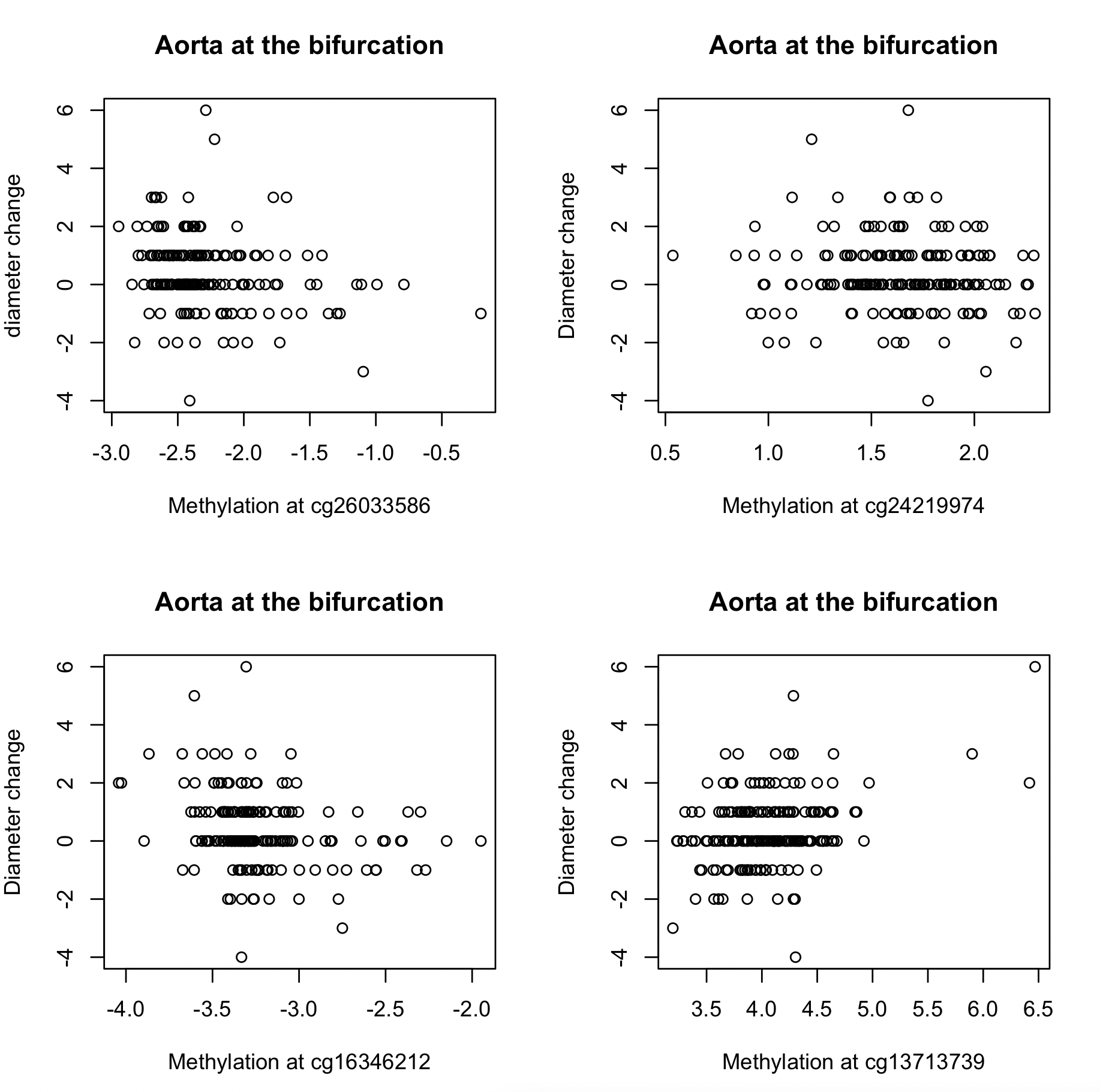


**ST1.** Normal range values in healthy controls.

|  | | |
| --- | --- | --- |
|  | | Healthy Controls |
| *Estimated cell fractions (%)* | | |
|  | B cells | 5 - 10 |
|  | NK cells | 10 - 30 |
|  | CD4+ T cells | 25 - 60 |
|  | CD8+ T cells | 5 - 30 |
|  | Neutrophils | 43 - 73 |
|  | Monocytes | 5 - 10 |
|  | Eosinophils | 0 - 5 |
